# Interdigitation-Induced Order and Disorder in Asymmetric Membranes

**DOI:** 10.1007/s00232-022-00234-0

**Published:** 2022-04-26

**Authors:** Moritz P. K. Frewein, Paulina Piller, Enrico F. Semeraro, Krishna C. Batchu, Frederick A. Heberle, Haden L. Scott, Yuri Gerelli, Lionel Porcar, Georg Pabst

**Affiliations:** 1 Institute of Molecular Biosciences, University of Graz, NAWI Graz, 8010 Graz, Austria; 2 Institut Laue-Langevin, 38042 Grenoble, France; 3 BioTechMed Graz, 8010 Graz, Austria; 4 Field of Excellence BioHealth, 8010 Graz, Austria; 5 Department of Chemistry, University of Tennessee, Knoxville, TN 37996 USA; 6 Center for Environmental Biotechnology, University of Tennessee, Knoxville, TN 37996 USA; 7 Shull Wollan Center, Oak Ridge National Laboratory, Oak Ridge, TN 37831 USA; 8 Department of Life and Environmental Sciences, Università Politecnica delle Marche, 60131 Ancona, Italy

**Keywords:** Asymmetric membranes, Transleaflet coupling, Cyclodextrin, Small-angle X-ray scattering, Small-angle neutron scattering

## Abstract

We studied the transleaflet coupling of compositionally asymmetric liposomes in the fluid phase. The vesicles were produced by cyclodextrin-mediated lipid exchange and contained dipalmitoyl phosphatidylcholine (DPPC) in the inner leaflet and different mixed-chain phosphatidylcholines (PCs) as well as milk sphingomyelin (MSM) in the outer leaflet. In order to jointly analyze the obtained small-angle neutron and X-ray scattering data, we adapted existing models of trans-bilayer structures to measure the overlap of the hydrocarbon chain termini by exploiting the contrast of the terminal methyl ends in X-ray scattering. In all studied systems, the bilayer-asymmetry has large effects on the lipid packing density. Fully saturated mixed-chain PCs interdigitate into the DPPC-containing leaflet and evoke disorder in one or both leaflets. The long saturated acyl chains of MSM penetrate even deeper into the opposing leaflet, which in turn has an ordering effect on the whole bilayer. These results are qualitatively understood in terms of a balance of entropic repulsion of fluctuating hydrocarbon chain termini and van der Waals forces, which is modulated by the interdigitation depth. Monounsaturated PCs in the outer leaflet also induce disorder in DPPC despite vestigial or even absent interdigitation. Instead, the transleaflet coupling appears to emerge here from a matching of the inner leaflet lipids to the larger lateral lipid area of the outer leaflet lipids.

**Graphical abstract:**

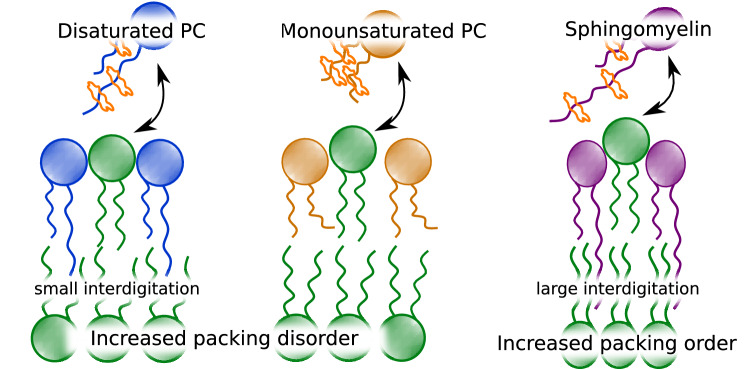

**Supplementary Information:**

The online version of this article (10.1007/s00232-022-00234-0) contains supplementary material, which is available to authorized users.

## Introduction

Plasma membranes play pivotal roles in cell physiological processes by regulating and controlling diverse signaling, sensing and transport mechanisms. One of the outstanding features of plasma membranes on the molecular level is a pronounced asymmetric distribution of its lipids across the bilayer, which is generated and controlled by proteins known as flipases, flopases and scramblases (van Meer 2011). Historically, lipid asymmetry was proposed for erythrocytes already half a century ago (Bretscher 1972), i.e. the same year the famous fluid-mosaic model for membrane structure was coined by Singer and Nicolson (1972). Only 1 year later Verkleij et al. reported first experimental data for lipid asymmetry in erythrocytes using a clever combination of lipid degrading enzymes (Verkleij et al. 1973). Most recently these data were confirmed also for other eukaryotes and extended to details of hydrocarbon chain asymmetry (Lorent et al. 2020). The emerging picture is that eukaryotic plasma membranes have an outer leaflet enriched in choline lipids, such as phophosphatidylcholines (PC) and sphingomyelins (SM), and an inner leaflet containing the amino lipids phosphatidylethanolamine and phosphatidylserine, as well as phosphatidylinositol.

There is yet another type of asymmetry. Most naturally occurring membrane lipids have mixed hydrocarbons. Phospholipids for example typically have a saturated hydrocarbon chain at the *sn*-1 position of the glycerol backbone and unsaturated hydrocarbon at *sn*-2. In eukaryotic plasma membranes the majority of these (poly)unsaturated hydrocarbons is located in the inner leaflet (Lorent et al. 2020). Mammalian sphingomyelin in turn has in general only few double bonds in its hydrocarbons, but significantly different chain lengths. Interestingly, lipidomics data on plasma membrane leaflet composition showed also small amounts ($\sim$ 1 mol %) of saturated chain asymmetric phosphatidylcholine, such as 14:0–16:0 PC, 14:0–18:0 PC and 16:0–18:0 PC (Lorent et al. 2020). While mixed saturated/unsaturated hydrocarbons have been related to a compromise between bilayer bending flexibility and permeability (Antonny et al. 2015), little is known about the role of mixed saturated phospholipids. Chiantia and London reported an effect of brain sphingomyelin and milk sphingomyelin (MSM) on the lateral diffusion of inner leaflet lipids using asymmetric lipid vesicles fabricated by cyclodextrin (CD)-mediated lipid exchange (Chiantia and London 2012). This type of coupling was found to depend on the extent of chain-length asymmetry, as well as the hydrocarbon chain composition of the inner leaflet lipids. That is, the lateral diffusion of dioleoyl PC was slowed down by MSM only, which was attributed to the longer interdigitating N-acyl chain of MSM. Surprisingly, fluorescence lifetime measurements of the systems found no effect of hydrocarbon chain interdigitation on the overall order of the inner leaflet lipids, demonstrating that interleaflet coupling can be different for different membrane properties. Hydrocarbon chain interdigitation-mediated ordering of lipids in the opposing leaflet was observed in molecular dynamics (MD) simulations, however (Róg et al. 2016).

In general, hydrocarbon chain interdigitation is thought to be an important factor in a functional coupling of both membrane leaflets even in the absence of proteins, although other mechanisms have also been discussed (see e.g. Eicher et al. 2018 and references therein). Interdigitation is believed to increase the shear viscosity between membrane leaflets, which appears to be consistent with the observed reduction of lateral diffusion discussed above (Chiantia and London 2012). This is contrasted, however by experiments with short and long chain fluorescent lipid analogues penetrating to different amounts into the opposing lipid leaflet, which did not reveal different interleaflet viscosities (Horner et al. 2013). We have recently reported the structure of 14:0–18:0 PC (MSPC), 18:0–14:0 PC (SMPC), 16:0–14:0 PC (PMPC) and MSM symmetric bilayers combining small-angle X-ray and neutron scattering (SAXS, SANS) and MD-simulations (Frewein et al. 2021). Indeed, we observed an increase of the extent of interdigitation with increasing length difference between the two chains. Interestingly, however, we also found that a significant fraction of the longer chain is bending back and hence not penetrating into the opposing leaflet. This indicates that effects of hydrocarbon ordering in the opposing leaflet might at least to some extent not originate from interdigitation and the associated interleaflet viscosity.

In order to gain further insight, we performed SAXS/SANS experiments on asymmetric large vesicles (aLUVs) with an inner leaflet composed of mainly di16:0 PC (DPPC) and outer leaflets enriched in either MSPC, SMPC, PMPC or MSM. In the following, these systems are referred to as DPPC$^\text {in}$/MSPC$^\text {out}$, DPPC$^\text {in}$/SMPC$^\text {out}$, DPPC$^\text {in}$/PMPC$^\text {out}$ and DPPC$^\text {in}$/MSM$^\text {out}$. This does not imply, however, complete lipid exchange. The advantage of SAXS/SANS experiments is the lack of bulky labels that might either perturb the delicate balance of intermolecular forces in bilayers or not sample all intramembraneous environments equally. This advantage is, however, frequently challenged by the need for extensive data modeling (Semeraro et al. 2021). We have previously reported models for analyzing scattering data of aLUVs (Heberle et al. 2016; Eicher et al. 2017). For the hitherto studied systems, containing 16:0–18:1 PC (POPC), 16:0–18:1 phosphatidylethanolamine, and DPPC, we observed a structural leaflet coupling only when at least one of the leaflets was in the gel phase, but not for all-fluid membranes, i.e. in the L$_\alpha$ phase (Heberle et al. 2016; Eicher et al. 2018). Here, we focus on fluid membranes using a modified asymmetry model, which features in addition to the recently introduced headgroup hydration layer (Frewein et al. 2021) also the possibility that the center of mass of the terminal methyl groups does not coincide with the center of the lipid bilayer. Such scenarios might occur due to hydrocarbon chain interdigitation or back-bending and are expected for the currently studied systems.

We found that all aLUVs with saturated chain asymmetric PCs have an increased area per lipid in both leaflets, i.e. decreased molecular packing, as compared to the same lipids, but in symmetric bilayers. Apparently, this results from only a minor interdigitation of the longer hydrocarbon chain into the opposing DPPC leaflet that presumably leads to an increase of configurational entropy of all lipid chains. In contrast we observed for DPPC$^\text {in}$/MSM$^\text {out}$ an about three times deeper interdigitation of the long MSM acyl chains and a concomitant lateral condensation of both lipid leaflets, suggesting a loss of hydrocarbon configurational entropy due to increased van der Waals forces. We additionally, applied our analysis to aLUVs with an outer leaflet enriched in the monounsaturated lipids POPC and 18:0–18:1 PC (SOPC), i.e., DPPC$^\text {in}$/POPC$^\text {out}$, and DPPC$^\text {in}$/SOPC$^\text {out}$. In this case, we find an increased area per lipid in both cases, however the direction of the shift of the methyl-groups is opposite in the cases. While back-bending of the 18:1-chains prevails for POPC, the longer 18:0-chains in SOPC slightly interdigitate into the DPPC-leaflet.

## Results and Discussion

### Modeling aLUVs with Chain Asymmetric Lipids

**Fig. 1:**
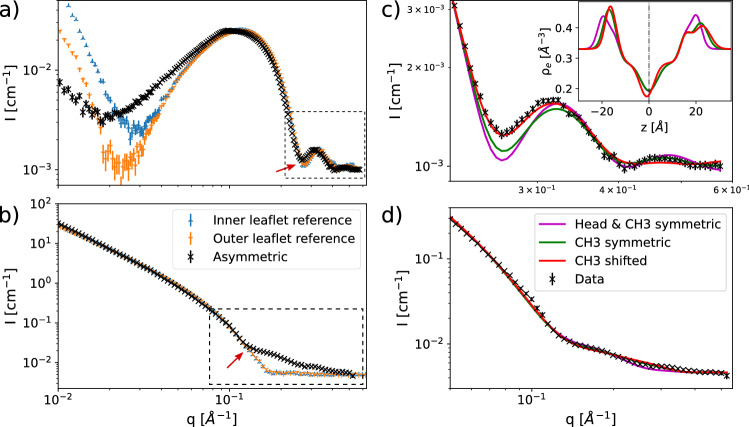
Comparison of SAXS (**a**) and SANS (100 % D$_2$O, **b**) curves of reference-LUVs and aLUVs consisting of DPPC (DPPC-d62 for aLUVs) and SMPC. Data have been rescaled for clarity. **c** Shows SAXS model fits of the window indicated with a dashed line in (**a**), with the corresponding electron density trans-bilayer profiles in the inset. SANS (100 % D$_2$O, **d**) data of DPPCd62$^\text {acc}$ SMPC$^\text {don}$ asymmetric vesicles and model fits (solid lines) using 3 different models with increasing asymmetry

Figure 1a and b show SAXS/SANS data of DPPC$^\text {in}$/SMPC$^\text {out}$ aLUVs in comparison to scattering data from symmetric LUVs composed of DPPC/SMPC mixtures representing either the inner or the outer leaflets of the aLUVs. Data have been obtained at 50$^{\circ }$C, i.e. well-above the chain melting temperatures of both lipids (Marsh 2013). SAXS data most clearly deviate at low scattering vectors, *q*. This indicates a modification of the lipid’s headgroup scattering contrast, e.g. due to differing hydration (Frewein et al. 2021). As discussed previously (Frewein et al. 2021), SANS is not sensitive to this effect because of the lower contrast in the headgroup regime. In the following, we focus specifically on the mid-*q* range, however, where SAXS and SANS data of aLUVs both show a pronounced ’lift-off’ of the scattered intensity as compared to the compositionally symmetric LUVs (dashed boxes in Fig. 1a, b). The degree the asymmetric curves lift off from the incoherent baseline in SANS is a known measure for the difference in deuteration between inner and outer leaflet that creates a contrast in the neutron SLD-profile in the hydrocarbon regime, see e.g. Eicher et al. 2017). This effect can also be used to monitor the stability of the system with respect to lipid flip–flop (Nguyen et al. 2019; Marx et al. 2021) (for stability checks for the presently studied systems, see Appendix B). To accentuate this lift-off in SANS, we used chain deuterated DPPC (DPPCd62) as acceptor lipid. For our here studied aLUVs this matches the scattering contrast between the inner leaflet hydrocarbons and the solvent in case of 100 % D$_2$O. Also SAXS data show an intensity lift-off in this *q*-range, which can, however, not be uniquely attributed to the asymmetric composition of the aLUVS. For example, a similar effect has been observed before for small unilamellar vesicles and interpreted as head group asymmetry due to an increased membrane curvature (Brzustowicz and Brunger 2005; Kučerka et al. 2007). However, even compositionally symmetric LUVs may show such features, which can be accounted for by considering membrane thickness fluctuations (Frewein et al. 2021).

To jointly analyze SAXS/SANS data of asymmetric vesicles with both leaflets in the L$_\alpha$ phase, we adopted the following strategy. Firstly, we modified the scattering density profile (SDP)-model for flat asymmetric bilayers (Eicher et al. 2017) with a vesicle form factor and a headgroup hydration layer as detailed recently for symmetric LUVs (Frewein et al. 2021). Compared to previous SAXS/SANS reports on aLUV structure this allowed us to include also low-*q* data in the analysis (Eicher et al. 2017, 2018). The modified SDP-model for aLUVs also included the above mentioned membrane thickness fluctuations (Frewein et al. 2021). However, the obtained fits yielded unsatisfying results (Fig. 1c). Inspired by (Brzustowicz and Brunger 2005; Kučerka et al. 2007), we therefore considered, in a second attempt, the possibility of headgroup asymmetry. That is, the volume distribution functions describing inner and outer leaflet phosphate groups (PCN) and choline-CH$_3$ groups were allowed to differ in their relative positions to the backbones, and also in the widths of the phosphate groups $\sigma _{PCN}^\text {in/out}$. This improved the agreement between model and data only slightly, however (Fig. 1c). In the third step we finally took into account that the longer hydrocarbon of the outer leaflet SMPC can interdigitate into the inner leaflet. In terms of our SDP-model this means that some of the terminal methyls will be off-centered from the interface between the lipid leaflets; for details see “SAS-Data Analysis” section and Appendix A. The obtained fits gave the overall best agreement with SAXS data (Fig. 1c). Note that differences between the three models are small in SANS (Fig. 1d). This supports the idea that the observed lift-off in SAXS is dominated by hydrocarbon interdigitation. We also tried to model data with interdigitated hydrocarbons, but symmetric heads. This decreased the agreement between model and data, however (data not shown). The final successful model therefore allows in addition to our previous model for aLUVs (Eicher et al. 2018) also for interdigitated hydrocarbons and asymmetric lipid heads. The origin of headgroup asymmetry for the here studied systems is unclear at present, but might be due to lipid crowding (mass imbalance) because of CD-mediated lipid exchange.

**Fig. 2:**
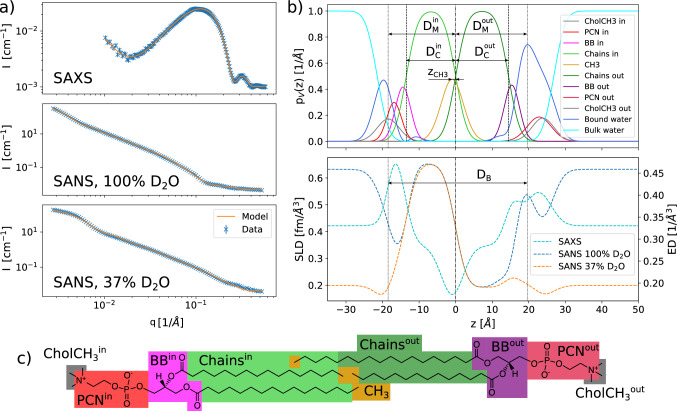
Model fits to full *q*-range SAXS and SANS data (**a**), using the asymmetric SDP- and separated form factor model. **b** Shows the SDP-model volume probability functions of lipid moieties and surrounding water and the resulting neutron-SLD and electron density (ED) profiles. The neutron-SLD of inner and outer leaflet chain regions differ greatly due to the inner leaflet enriched in chain-deuterated DPPCd62. The parsing scheme for PCs is shown in **c**, using the corresponding colors to the SDP model functions

Figure 2a demonstrates the excellent agreement of our model for DPPC$^\text {in}$/SMPC$^\text {out}$ aLUVs over the complete studied *q*-range. These data also include SANS measurements performed at 37% D$_2$O. At this contrast the solvent roughly matches the outer leaflet of our vesicles, which mostly contains protiated lipids. A combination with other contrasts gives therefore additional constraints for the adjustable parameters of our model. The applied parsing of membrane structure with volume distribution functions and resulting electron and neutron scattering density profiles are presented in Fig. 2b and c. Figure 2b also shows several parameters used to describe the transmembrane structure. These include the Luzzati bilayer thickness $D_B$, as the sum of the inner and outer monolayer thicknesses, $D_M^\text {in}$, $D_M^\text {out}$, the leaflet thicknesses of the hydrocarbons, $D_C^\text {in}$, $D_C^\text {out}$, as well as the position of the center of the terminal CH$_3$ distribution function, $z_\text {CH3}$. Due to the ability of hydrocarbons to also bend back, $z_\text {CH3}$ measures interdigitation and back-bending at the same time. For details and all definitions see “ SAS-Data Analysis” section and Appendix A. The results for the structure of DPPC$^\text {in}$/SMPC$^\text {out}$ aLUVs are listed in Table 1 and will be discussed in the next section; all adjustable parameters for the fits are reported in Supplementary Table S2.

### Structure of aLUVs with Saturated Chain Asymmetric Lipids

Having established a structural model for the analysis of asymmetric vesicles containing chain asymmetric lipids we first applied it to aLUVs with outer leaflets enriched in MSPC, SMPC and PMPC, while maintaining DPPC based inner leaflets. Results listed in Table 1 report our best fit-results for the main compositional and structural parameters (full details are given in Tables S2 and S3; for fits see Figs. 2, S2 and S3). $\chi _\text {acc/don}$ designate the acceptor/donor concentrations in the vesicles, which we constrained during the modeling by the results of the compositional analysis using gas chromatography (see “Compositional Analysis Using Gas Chromatography” section and Supplement). The distribution over both leaflets is given by $\chi ^\text {in/out}_\text {acc/don}$ and results from the analysis of SANS-data, as discussed in “Modelling aLUVs with Chain Asymmetric Lipids” section. The lipid exchange efficiencies yielded an about 70% exchange of the outer leaflet and varied only slightly between the different samples. Only for MSPC this was reduced to 55%. Regarding structural parameters, the Luzzati thickness $D_B$ is a well established measure for the width of a bilayer, taking into account its smeared out water/bilayer interface (Tristram-Nagle 2015). Our measure for the interdigitated state of the hydrocarbon chains is the position of the center of the volume probability distribution of the terminal methyl groups with respect to the bilayer center $z_\text {CH3}$. Negative values imply a shift towards the inner leaflet and *vice versa*. We introduced the parameters $V_\text {bw}$ and $\sigma _\text {poly}$ in Frewein et al. (2021) to describe the volume per bound water molecule and the membrane thickness polydispersity. We do not discuss these results in further detail, as the exact nature of this polydispersity is still unclear and $V_\text {bw}$ is strongly coupled to the total number of bound water molecules, $n_W$. The numbers we find here for $V_\text {bw}$ and $\sigma _\text {poly}$ agree quite well with the ones observed for symmetric vesicles (Frewein et al. 2021). We therefore expect no fundamental differences in these properties between symmetric and asymmetric vesicles. $D_M^\text {in/out}$ are the monolayer thicknesses and calculated equivalently to $D_B$, but separately for each leaflet. In case of the references, $D_M^\text {in/out}$ is $D_B/2$ of the respective vesicle and $D_B$ is the sum of both half-bilayer thicknesses. The hydrophobic thicknesses $D_C^\text {in/out} = V^\text {in/out}_\text {HC}/A^\text {in/out}$ are connected to the area per lipid of the respective leaflet $A^\text {in/out}$ by the chain volumes $V^\text {in/out}_\text {HC}$, for which we used tabulated values depending on the number of hydrocarbons in the lipid (Nagle et al. 2019). To get a feeling how the structure compares to the one of its components, we also calculated the area for each lipid $A_\text {acc/don}$, by assuming linear additivity of the areas: $A^\text {in/out}=\chi _\text {acc}^\text {in/out}A_\text {acc}+\chi _\text {don}^\text {in/out}A_\text {don}$. The structure of the lipid headgroup is determined by several adjustable parameters, which are further discussed in section A. Here we give the number of water molecules per headgroup within the Luzzati thickness $n_W^\text {in/out}$, which reflects the extension of the headgroup into the aqueous phase. In symmetric LUVs of chain-asymmetric PCs values for $n_W$ between 9 and 15 were found (Frewein et al. 2021).

The transbilayer structures of symmetric DPPC, MSPC, SMPC and PMPC bilayers in the L$_\alpha$ phase have been recently shown to be relatively similar, with the most striking difference being the localization of the CH$_3$-groups, which exhibits a linear dependence on the chainlength-mismatch (Frewein et al. 2021). In particular, the lateral area per lipid of these lipids was between 62 and 63 Å$^2$, suggesting that there are no significant perturbations in the bilayer caused by chain-mismatch. Also symmetric mixtures of either of the chain-asymmetric lipids with DPPC, which were prepared as symmetric references mimicking either the inner or the outer leaflet of the aLUVs, showed no significant differences in structure; structural parameters for these reference samples are given in Table 1 (see also Tables S1 and S3; Figs. S4 and S5).

In the case of asymmetric vesicles, however, the presence of a chain-asymmetric lipid in the outer leaflet leads to a shift of the terminal methyl groups towards the DPPC-containing (inner) leaflet. We note the high experimental uncertainty of the absolute values of CH$_3$ location (Table 1), which impedes us from discussing differences between MSPC, SMPC, and PMPC. A common observation for all three lipids is that the area per lipid in the inner leaflet is increased, for MSPC and PMPC also in the outer leaflet. This suggests that hydrocarbon chains, which penetrate into an opposing leaflet of lipids with less chainlength-mismatch, evoke a state of higher disorder in the hydrocarbon chain region. Notably, there are also changes in the headgroup regions, which seem to emerge along with the CH$_3$-asymmetry. Outer leaflet headgroups extend further into the water and therefore accommodate a higher number of water molecules than in the symmetric references. Overall compared to membranes of symmetric inner and outer leaflet references these asymmetric bilayers are thinner, as a result of the increase of area per lipid in one or both leaflets.

Next we focused on the natural lipid mixture MSM, which comprises a much higher chain-asymmetry than the here studied PCs due to its prevalent long acyl chains (22:0, 23:0, 24:0, 24:1). In symmetric vesicles of MSM we found a wider spread of the CH$_3$-region than for all studied PCs and a slightly higher area per lipid of 64.8 Å$^2$ (Frewein et al. 2021). Inner and outer leaflet mixtures of MSM and DPPC have $A^\text {in} \sim 64$ Å$^2$ and $A^\text {out} \sim 63$ Å$^2$ in symmetric LUVs (Tables 1 and S4; Fig. S6). In asymmetric vesicles the area per lipid of the inner leaflet is $\sim 5$% smaller than in the reference, with about twice as much interdigitating hydrocarbons as compared to aLUVs with the donors MSPC and SMPC. The long chains of MSM seem to have an ordering effect on DPPC, which is neither present in symmetric vesicles, nor in asymmetric vesicles with less interdigitation. In contrast, outer leaflet MSM has a similar effect on the headgroup regions of the asymmetric vesicles compared to outer leaflet MSPC, SMPC, and PMPC, i.e., the outer leaflet of the asymmetric vesicle is more hydrated than the inner leaflet.

Given that for fluid phase asymmetric vesicles there is not yet any evidence that opposing monolayers would influence each others structures (Eicher et al. 2017, 2018) and that interdigitation has been shown to have little to no influence on lipid diffusion in symmetric bilayers (Schram and Thompson 1995; Horner et al. 2013), this is a surprising result in asymmetric bilayers. Our data suggest a delicate interplay between repulsive entropic/steric forces and attractive van der Waals interactions. In asymmetric bilayers with low interdigitation, the configurational entropy contribution of the hydrocarbon termini apparently dominate and the penetrating chain segments perturb the packing of the opposing DPPC. In contrast, the long chains of MSM share a larger surface of contact and their cohesion leads to an ordering of chains. Indeed, MD-simulations of asymmetric membranes containing 24:0-sphingomyelin reported an increase of order for the interdigitating moieties of its hydrocarbon chain (Róg et al. 2016).

**Table 1:** Fit results for saturated mixed-chain PCs and MSM as well as properties of inner/outer leaflet reference LUVs. $\epsilon$ is the error for all aLUV parameter values

Donor lipid	$\epsilon$ [%]	MSPC		SMPC		PMPC		MSM	
		aLUV	Ref	aLUV	Ref	aLUV	Ref	aLUV	Ref
$\chi _{acc}:\chi _{don}$ %	5	69:31		59:41		62:38		54:46	
$\chi _{acc}^{in}:\chi _{don}^{in}$ %	5	96:04		92:08		95:05		83:17	
$\chi _{acc}^{out}:\chi _{don}^{out}$ %	5	45:55		30:70		33:67		28:72	
$D_B$ [Å]	3	37.0	39.6	38.1	39.5	36.1	38.6	40.9	40.0
$z_{CH3}$ [Å]	10	− 0.96		− 0.95		− 0.69		− 2.59	
$V_{bw}$ [Å$^{3}$]	6	29.4		29.3		29.6		29.5	
$\sigma _{poly}$ [%]	6	4.1		2.8		3.3		7.3	
$D_M^{in}$ [Å]	6	18.0	19.8	18.5	19.8	18.0	19.3	20.0	19.4
$D_M^{out}$ [Å]	6	18.9	19.8	19.6	19.7	18.1	19.3	21.0	20.6
$D_C^{in}$ [Å]	5	13.2	14.5	13.5	14.6	13.2	14.1	14.8	14.3
$D_C^{cout}$ [Å]	5	14.0	14.5	14.5	14.4	13.2	14.0	16.4	16.1
$A^{in}$ [Å$^{2}$]	5	67.4	62.4	65.8	62.1	67.5	63.7	60.9	64.2
$A^{out}$ [Å$^{2}$]	5	65.8	62.2	63.5	62.7	66.5	61.7	63.9	63.2
$A^{acc}$ [Å$^{2}$]	5	67.6	63.1$^\dagger$	66.1	63.1$^\dagger$	67.6	63.1$^\dagger$	60.0	63.1$^\dagger$
$A^{don}$ [Å$^{2}$]	5	64.4	62.2$^\dagger$	62.4	62.0$^\dagger$	66.0	62.9$^\dagger$	65.4	64.8$^\dagger$
$n_W^{in}$	6	9.5	6.4	6.5	6.8	9.0	12.5	8.0	7.8
$n_W^{out}$	6	15.7	4.6	15.2	5.5	15.8	4.5	11.1	6.8

$^{*}$Reference values from symmetric inner/outer leaflet references (Figs. S4–S6; Tables S3 and S4)

$^\dagger$ From single lipid references (Table S1)

### Structure of aLUVs with Monounsaturated Lipids

Finally, we extended our study also to aLUVs with outer leaflets enriched in POPC or SOPC, i.e. lipids with mixed saturated and unsaturated hydrocarbons. Such mixed chain lipids are much more common in mammalian plasma membranes than those studied in the previous section (Lorent et al. 2020). Interestingly, these lipids share with PMPC, SMPC, and MSPC, a nearly equal overlap region of interdigitating and back-bending terminal methyl groups in symmetric bilayers (Frewein et al. 2021). In the case of asymmetric membranes the role of unsaturated hydrocarbons at the bilayer center is even less clear. We have therefore employed the here developed analysis model also to DPPC$^\text {in}$/POPC$^\text {out}$ and DPPC$^\text {in}$/SOPC$^\text {out}$ aLUVs.

Table 2 gives the corresponding results for the main structural parameters (see also Table S4; Fig. S3). The overall lipid exchange was about equal for both lipids, although POPC flipped slightly more into the inner leaflet during aLUV preparation. The overall membrane thickness $D_B$ of both aLUVs was about 2 Å thinner than the combined $D_B/2$-values of symmetric inner and outer leaflet mimics. Within experimental uncertainty these thickness changes occurred almost equally in both leaflets, i.e. $\Delta D_M^\text {in} \sim \Delta D_M^\text {out}$, although the inner leaflets thickness appears to be somewhat more affected. This is particularly reflected in the changes of $D_C^\text {in}$ and $D_C^\text {out}$, which were more pronounced for the DPPC-enriched inner leaflet. This leads to a large increase of $A^\text {in}$ by about 8% as compared to symmetric DPPC bilayers, or a change of DPPC lateral area $\Delta A^\text {acc}/A^\text {acc} \sim 7\%$. The area per lipid values in the outer leaflet remained unchanged within experimental uncertainty. Moreover, the areas per lipid in the inner leaflet closely matched those of the outer leaflet.

Analysis for the position of the terminal CH$_3$ center of mass revealed interesting differences between POPC and SOPC containing aLUVs. In particular, we found that in the case of a POPC-donor, the CH$_3$function is located in the outer leaflet and resides for SOPC slightly within the inner leaflet. This could mean that, the 18:1-chains in the leaflet containing POPC bends back to match the length of 16:0. Another possibility is that the kink induced by the double bond of the 18:1-chains is responsible for the terminal methyls being located further in the outer leaflet. SOPC in turn, with its longer 18:0 chain seems to slightly interdigitate, thus shifting the CH$_3$ center of mass again into the DPPC-rich inner leaflet. The headgroups in both systems also exhibit a high degree of asymmetry. In the SOPC-rich leaflet they stretch to the outside, as it happens in the saturated systems, leading to a high number of water molecules per headgroup in the outer leaflet. For DPPC$^\text {in}$/POPC$^\text {out}$ this situation is reversed, showing a broader headgroup with more water in the inner leaflet. The symmetric reference vesicles qualitatively mirror this behavior, with the outer leaflet mimics having a higher number of hydration waters than the inner ones, however not to the extent of the asymmetric vesicles.

While understanding the subtle effects on headgroup hydration encourage additional experiments, the loosening of lipid packing in the inner leaflet of the presently studied aLUVs containing monounsaturated lipids emerges as a salient feature. Previous SANS experiments on POPC$^\text {in}$/DPPC$^\text {out}$ experiments also reported a softening of the DPPC-enriched leaflet below the melting temperature of DPPC, but no coupling effects when both leaflets were in the L$_\alpha$ phase (Heberle et al. 2016). Also in a later report, using joint SAXS/SANS experiments, we found no coupling for fluid POPC$^\text {in}$/DPPC$^\text {out}$ aLUVs (Eicher et al. 2017). We tested the parameters used in that study for our vesicles and indeed found another local minimum with the same areas per lipid. However, this drove the system to $z_\text {CH}3 = -\,2.5$ Å, which would imply a similar interdigitation into the inner leaflet as MSM. We therefore consider this solution less likely. We speculate that absence of interleaflet coupling in our previous studies is a combination of lower data quality, and improved modeling and optimization routines used for fitting present data. However, we cannot fully exclude sample specific properties or slight differences in sample preparation as a potential cause.

**Table 2:** Fit results for aLUVs with POPC and SOPC as donor lipids, as well as properties of inner/outer leaflet reference LUVs

Donor lipid	$\epsilon$ [%]	POPC		SOPC	
		aLUV	Ref	aLUV	Ref
$\chi _\text {acc}:\chi _\text {don}$ %	5	64:36		61:39	
$\chi _\text {acc}^\text {in}:\chi _\text {don}^\text {in}$ %	5	85:15		93:07	
$\chi _\text {acc}^\text {out}:\chi _\text {don}^\text {out}$ %	5	45:55		33:67	
$D_B$ [Å]	3	36.3	38.9*	37.3	39.3*
$z_\text {CH3}$ [Å]	10	1.00		$-\,$0.35	
$V_\text {bw}$ [Å$^{3}$]	6	29.60		29.6	
$\sigma _\text {poly}$ [%]	6	4.5		6.1	
$D_M^\text {in}$ [Å]	6	17.8	19.7*	18.2	20.0*
$D_M^\text {out}$ [Å]	6	18.6	19.2*	19.1	19.4*
$D_C^\text {in}$ [Å]	5	13.0	14.5*	13.4	14.7*
$D_C^{cout}$ [Å]	5	13.8	14.2*	14.3	14.5*
$A^\text {in}$ [Å$^{2}$]	5	68.9	62.8*	67.7	62.3*
$A^\text {out}$ [Å$^{2}$]	5	68.3	65.9*	68.5	67.3*
$A^\text {acc}$ [Å$^{2}$]	5	67.4	63.1$^\dagger$	67.6	63.1$^\dagger$
$A^\text {don}$ [Å$^{2}$]	5	68.9	67.5$^\dagger$	69.0	68.8$^\dagger$
$n_W^\text {in}$	6	17.9	11.0*	9.0	7.2*
$n_W^\text {out}$	6	9.0	4.9*	20.4	14.6*

$\epsilon$ is the error for all aLUV parameter values

$^{*}$ Reference values from symmetric inner/outer leaflet references (Figs. S6 and S7; Table S4)

$^\dagger$ From single lipid references (Table S1)

## Conclusion

**Fig. 3:**
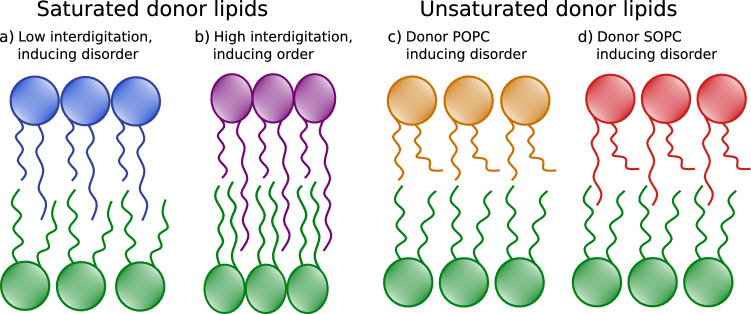
Schematic of possible lipid arrangements of interdigitated systems with saturated lipids of low(**a**) (DPPC$^\text {in}$/MSPC$^\text {out}$, DPPC$^\text {in}$/SMPC$^\text {out}$, DPPC$^\text {in}$/PMPC$^\text {out}$) and high (**b**) chainlength-mismatch (DPPC$^\text {in}$/MSM$^\text {out}$), as well as DPPC$^\text {in}$/POPC$^\text {out}$ (**c**) and DPPC$^\text {in}$/SOPC$^\text {out}$ (**d**)

To the best of our knowledge the present study provides the first experimental evidence of structural coupling of all-fluid asymmetric bilayers. Previously, transbilayer coupling was only observed when at least one leaflet was in the gel phase (Heberle et al. 2016; Eicher et al. 2018), including also lateral diffusion studies of chain asymmetric sphingomyelin (Chiantia and London 2012). The observed coupling suggests a subtle balance of ordering and disordering effects at the hydrophobic interface between the two leaflets as schematically shown in Fig. 3. For MSPC, SMPC, PMPC we found a minor interdigitation, which led to a loosening of the packing of inner leaflet DPPC. MSM, whose long acyl chains penetrates significantly into the opposing monolayer instead caused an overall lateral condensation of the bilayer. We propose that the configurational entropy of the hydrcarbons, which increases with chain length is able to disorder by fluctuation-mediated steric repulsion the inner lipid leaflets only upon minor chain overlap. On the contrary, energetic optimization of hydrocarbon cohesive forces outweighs this effect in the case of large interdigitation. Such a scenario indeed was suggested from MD simulations (Róg et al. 2016).

The decrease of inner leaflet DPPC packing in the case of outer leaflets enriched in POPC and SOPC suggest an additional scenario. Here, the larger lateral area required by the unsaturated hydrocarbon seems to generate a packing mismatch, which is alleviated by increasing the area per lipid of DPPC residing in the inner leaflet. Both scenarios are likely to affect differential stress between the two leaflets (Hossein and Deserno 2020). As most chain-asymmetric saturated lipids are long-chain sphingomyelins such as the ones used in this study or phospholipids with one mono or polyunsatured hydrocarbon chain, such transleaflet coupling schemes might indeed be present also in natural membranes. We note, however, that cholesterol, which is the most abundant lipid in mammalian plasma membranes has been shown to modulate interdigiation-based ordering of inner leaflet lipids (Róg et al. 2016). In order to keep our analysis tractable, we had to exclude cholesterol in the present study. With ongoing efforts in fabricating and analyzing more realistic models of mammalian plasma membranes such goals seem within reach.

## Methods

### Lipids, Chemicals and Sample Preparation

Lipids were purchased from Avanti Polar Lipids (Alabaster, AL, USA) and used without further purification. Chloroform, methanol (pro analysis grade), sucrose and methyl-$\beta$-cyclodextrin (m$\beta$CD) were obtained from Merck KGaA, Darmstadt, Germany. We prepared asymmetric unilamellar vesicles following the heavy donor cyclodextrin exchange protocol (Doktorova et al. 2018). Acceptor and donor lipids were weighed (ratio 1:2 mol/mol), dispersed separately in a chloroform/methanol mixture (2:1, vol/vol) and dried under a soft argon beam in a glass vial. Acceptor vesicles were prepared from a mixture (19:1 mol/mol) of chain deuterated DPPC (DPPCd62) and dipalmitoyl phosphatidylglycerol (DPPGd62); donor vesicle consisted of the single species indicated. The resulting films were kept over night in vacuum to ensure the evaporation of all solvent and hydrated with ultrapure H$_2$O containing 25 mM NaCl (acceptors, 10 mg/ml lipid) or 20% (wt/wt) sucrose (donors, 20 mg/ml) followed by 1 h incubation at 50 °C (room temperature for POPC and SOPC) and 5 freeze/thaw cycles. Acceptor vesicles were extruded at 50 °C using a handheld mini extruder (Avanti Polar Lipids, AL, USA) with a 100 nm pore diameter polycarbonate filter 31 times or until reaching a polydispersity index < 10% (measured by dynamic light scattering (DLS) using a Zetasizer NANO ZS90, MalvernPanalytical, Malvern, UK).

Donor vesicles were diluted 20-fold with water and centrifuged at 20,000 g for 30 min. The supernatant was discarded, the resulting pellet suspended in a 35 mM m$\beta$CD solution (lipid:m$\beta$CD 1:8 mol/mol) and incubated for 2 h at 50 °C, while being shaken at a frequency of 600 min$^{-1}$. Acceptor vesicles were added and incubated for another 15 min. The exchange was stopped by diluting the mixture 8-fold with water and centrifuging again at 20,000 g for 30 min. The supernatant containing the asymmetric vesicles was then concentrated to < 500 $\mu$l using 15 ml-Amicon centrifuge filters (Merck, 100 kDa cut-off) at 5000 $\times$ *g*. To remove residual CD and sucrose, the filters were filled with the desired solvent (H$_2$O for SAXS and 37% and 100% D$_2$O for SANS) and re-concentrated in 3 cycles. The final vesicle-sizes were again measured by DLS to ensure the absence of donor MLVs.

Symmetric reference vesicles were prepared using only protiated lipids and extruded as the acceptor vesicles, but using pure H$_2$O or D$_2$O. Inner leaflet mimics contained 90 mol% acceptor lipid (DPPC/DPPG 19:1 mol/mol), 10 mol% donor lipid; the outer leaflet samples were mixtures of 30 mol% acceptor and 70 mol% donor lipid.

### Small-Angle Scattering (SAS) Experiments

SANS measurements were performed at D22, Institut Laue-Langevin, Grenoble, France, equipped with eiter 1 (DOI: 10.5291/ILL-DATA.9-13-822, DOI: ILL-DATA.TEST-3063) or 2 (DOI: 10.5291/ILL-DATA.9-13-938 ) $^3$H multidetectors. Sample-to-detector distances were 1.6, 5.6 and 17.8 m with corresponding collimations of 2.8, 5.6 and 17.8 m for the single detector setup; or 5.6 and 17.8 m with the second detector out of center at 1.3 m, with 5.6 and 17.8 m collimations. The neutron wavelength was 6 Å  ($\Delta \lambda /\lambda = 10 \%$). Samples were filled in Hellma 120-QS cuvettes of 1 mm pathway and heated to 50 °C using a bath circulator. Lipid concentrations were about 5 mg/ml in 100% D$_2$O and 15 mg/ml in 37% D$_2$O. Data were reduced using GRASP (www.ill.eu/users/support-labs-infrastructure/software-scientific-tools/grasp/ accessed on 25 June 2019), performing flat field, solid angle, dead time and transmission correction, normalizing by incident flux and subtracting contributions from empty cell and solvent.

SAXS data were recorded at BM29, ESRF, Grenoble, France (DOI: 10.15151/ESRF-ES-514136943), equipped with a Pilatus3 2 M detector, using a photon energy of 15 keV at a sample-to-detector distance of 2.867 m (Pernot et al. 2018). Samples were measured at a concentration of 10 mg/ml, at 50 °C and exposed for 20 times 2 s in a flow-through quartz capillary of 1 mm light path length. Data reduction and normalization were done by the automated ExiSAXS system; for subtraction of solvent and capillary contributions SAXSutilities 2 (www.saxsutilities.eu accessed on 29 October 2020) was used.

### SAS-Data Analysis

To analyze SAS-data we model our lipid bilayer using volume probability distribution functions describing the localization and extent of different parts of the lipids within the membrane. This approach has been previously introduced for SAS data evaluation as the SDP-model (Kučerka et al. 2008) and later extended to asymmetric bilayers (Eicher et al. 2017). For symmetric vesicles we use a previously introduced (Frewein et al. 2021) modified version of the SDP-model, which includes the vesicle form factor via the separated form factor-method (Pencer et al. 2006), membrane polydispersity and a headgroup-hydration shell. We also extend the asymmetric SDP-model by the same aspects, as well as modifying the distribution function of the terminal methyl to better allow for examining hydrocarbon chain interdigitation. The full model is presented in Appendix section A. To take into account the presence of lipid mixtures, we average all volumes and scattering lengths for each part of the lipid. To give an example, for a 1:1 mixture of DPPC and PMPC we assume an average lipid with 31 hydrocarbons. This includes the assumption that the lipids mix homogeneously within their leaflet. We note that the disagreement between model and low-*q* SAXS data for symmetric vesicles (Figs. S4–S7) is due to technical issues that occurred during the experiments, not to inadequacies of the model. Previously reported SAXS data of symmetric LUVs were fully accounted for in this *q*-range by including a hydration shell (Frewein et al. 2021). Moreover, we also showed that the hydration shell does not contribute to higher scattering vectors. Consequently, reported structural data for symmetric reference LUVs are not affected by difficulties in fitting low-*q* SAXS data.

Fitting was done as described in Frewein et al. (2021), including the same SAXS/SANS-weighting, negative water-penalty and Trust Region Reflective optimization algorithms (Virtanen et al. 2020). Errors were estimated from the covariance matrix, considering also possible systematic errors (e.g. from aLUV compositional uncertainties). For derived quantities we used Gaussian error propagation. For asymmetric vesicles we constrained the areas per lipid $A_\text {acc}$ and $A_\text {don}$ by Gaussian priors with mean and standard deviations of the results in Frewein et al. (2021). Also the total lipid concentrations $\chi _\text {acc}/\chi _\text {don}$ were constrained by Gaussian priors, using the results from gas chromatography (GC) compositional analysis. As the number of parameters describing the transmembrane structure is doubled, compared to symmetric vesicles, we fixed the distance between the hydrophobic interfaces and the backbones $d_\text {BB}$ to 0.9 Å  and the backbone width $\sigma _\text {BB}$ to 2.1 Å. Like for symmetric vesicles, we fixed the volumes of the individual moieties of the lipids according to Nagle et al. (2019) and the smearing parameters were set to $\sigma _\text {CH2} = 2.5$ Å and $\sigma _\text {Chol} = 3$ Å.

### Compositional Analysis Using Gas Chromatography

Fatty acid methyl esters (FAMEs) were prepared upon incubation with a Methanolic-H$_2$SO$_4$ solvent mixture and performed GC measurements using a GC 2010 Plus (Shimadzu), equipped with a split/splitless injector and a SGE BPX70-Cyanopropyl Polysilphenylene-siloxane column (25 m by 0.22 mm ID and 0.25 m film thickness) as described in Marx et al. (2021). For the calibration curves of 14:0, 18:0 and 18:1 hydrocarbon chains, we performed the same protocol with a concentration series with 1,2-dimyristoyl[1]sn-glycero-3-phosphocholine, 1,2-distearoyl-sn-glycero-3-phosphocholine and 1,2-dioleoyl-sn-glycero-3-phosphocholine, purchased from Avanti Polar Lipids (Alabaster, AL, USA ). Calibration curves and measurements can be found in the Supplementary Information Tables S5 and S6; Fig. S8.

### Supplementary Information

Below is the link to the electronic supplementary material.

Supplementary file1 (PDF 1286 kb)

## Figures and Tables

**Fig. 4:**
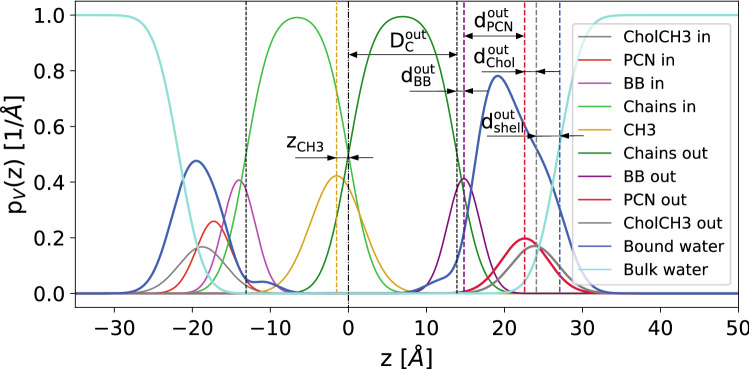
SDP-model and parameters used in the membrane form factor. Inner leaflet parameters are defined analogously

**Fig. 5:**
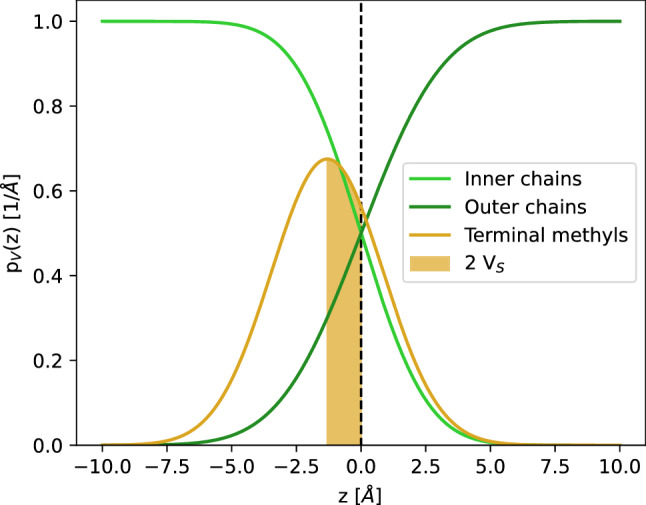
Arrangement of the hydrocarbon chain volume probability distributions in the membrane center. The distribution of CH3-groups (yellow) is assumed to shift to the left (inner leaflet), causing a transfer of volume $V_s$ from the outer to the inner leaflet. As the yellow curve comprises the methyl groups of both inner and outer leaflet, the shaded area corresponds to twice this volume

**Fig. 6:**
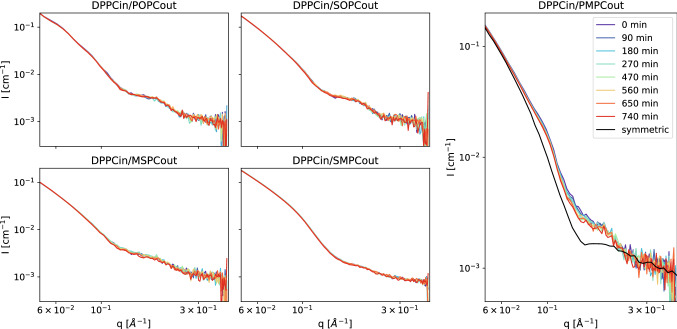
SANS-data of asymmetric vesicles monitored over 12 h at 50 °C. For DPPC$^\text {in}$/PMPC$^\text {out}$ the inner leaflet mimic is given as a reference

## Data Availability

SANS data (DOI: 10.5291/ILL-DATA.9-13-822, DOI: ILL-DATA.TEST-3063, DOI: 10.5291/ILL-DATA.9-13-938); SAXS data (DOI: 10.15151/ESRF-ES-51413694)

## Asymmetric SAS-Model

Models for asymmetric flat bilayers distinguish themselves from symmetric models mainly by the necessity to include the imaginary part of the form factor. We adopted the SDP model for asymmetric bilayers introduced in Eicher et al. (2017), with the following modifications: We included the vesicle form factor by the separated form factor method (Pencer et al. 2006) to describe low-*q* SANS data. We introduced some flexibility in the headgroup contrast using a higher-density hydration layer as described in Frewein et al. (2021). Finally, we described the terminal methyl groups of all lipids by a single error-function which is not necessarily centered around the bilayer center.

The necessary Fourier-transforms for the used distributions are given in the following. The error-function slab is centered around $\mu$, has a width of *d*, a smearing parameter of $\sigma$ and its area normalized to 1.

1 $$p_{{{\text{erf}}}} (z) = \frac{1}{{2d}}\int\limits_{{ - \infty }}^{\infty } {\left[ {{\text{erf}}\left( {\frac{{z - \mu + d/2}}{{\sqrt 2 \sigma }}} \right)\, - \,{\text{erf}}\left( {\frac{{z - \mu - d/2}}{{\sqrt 2 \sigma }}} \right)} \right]} {\text{e}}^{{iqz}} {\text{d}}z\, = \,\frac{{{\text{sin}}(qd/2)}}{{qd/2}}{\text{e}}^{{ - \frac{{\sigma ^{2} q^{2} }}{2}}} [\cos (\mu q) + i\sin (\mu q)]$$

For the Gaussian distribution centered at $\mu$ and standard deviation $\sigma$ we use:

2 $$p_{{{\text{Gauss}}}} (z) = \int\limits_{{ - \infty }}^{\infty } {\frac{1}{{\sqrt {2\pi } \sigma }}} {\text{e}}^{{ - \frac{{(x - \mu )^{2} }}{{2\sigma ^{2} }}}} {\text{e}}^{{iqz}} {\text{d}}z{\text{ }}\, = \,{\text{e}}^{{ - \frac{{q^{2} \sigma ^{2} }}{2}}} [\cos (\mu q) + i\sin (\mu q)]$$

**Table 3:** Molecular groups described by individual functions

Abbr.	Content	Function
*CH*3	Terminal methyl group	Error-function
*CH*2	Methylene chains	Error-function
*BB*	Backbone*	Gaussian
*PCN*	Phosphate + CN	Gaussian
*Chol*	Choline-CH3 group	Gaussian
*BW*	Hydration layer	Error-function

*Carbonyl-glycerol or sphingosine

We calculate the form factor by summing over the parts given in Table 3 for inner and outer leaflet, using the functions reported in Eqs. (1) and (2), multiplied by the respective weighting and contrast. Weighting factors for the headgroup contributions (*BB*, *PCN*, *Chol*) are $\frac{V_k}{A}$, *A* denoting the area per lipid and $V_k$ the volume of the respective moiety. All terminal methyl groups are condensed into one error function, whose center can deviate from the membrane center. This allows the description of interdigitated or back-bent states without increasing the number of parameters. Finally, *CH*2 and *BW* are weighted by the respective chain widths $D_C^\text {in/out}$, to fill the whole unit cell area, followed by subtraction of the quasi molecular groups they contain. In the case of an asymmetric *CH*3-distribution, however, the contribution of the methyl groups are not necessarily contained in the respective *CH*2-distributions. In this case, one of the leaflets loses some material with the volume $V_{s}$, which has to be respected in the model.

The connection between chain widths, volumes and area per lipid is then given by the following relations:

3 $$\begin{gathered} D_{C}^{{{\text{in}}}} = \frac{{(V_{{{\text{CH2}}}}^{{{\text{in}}}} + V_{{{\text{CH3}}}}^{{{\text{in}}}} + V_{s} )}}{{A^{{{\text{in}}}} }} \hfill \\ D_{C}^{{{\text{out}}}} = \frac{{(V_{{{\text{CH2}}}}^{{{\text{out}}}} + V_{{{\text{CH3}}}}^{{{\text{out}}}} - V_{s} )}}{{A^{{{\text{out}}}} }} \hfill \\ \end{gathered}$$

We define $V_s$ as half the integral over the terminal methyl group distribution function from its center $\mu _\text {CH3}$ to the center of the bilayer (the area indicated in Fig. 5), multiplied by the area per lipid of the leaflet towards which the distribution shifts:

4 $$V_{s} \, = \,\frac{{A^{{{\text{in/out}}}} }}{{2\int_{{\mu _{{{\text{CH3}}}} }}^{0} {p_{{{\text{CH3}}}} } (z){\text{d}}z}}$$

Note that the sign of $V_s$ changes according to the direction of the shift, being positive in case of a shift towards the inner leaflet and *vice versa*.

The membrane thickness polydispersity is implemented as described in Frewein et al. (2021), applying the same relative Gaussian distribution on inner and outer chain-width. Contrasts of the individual moieties *k* are defined by $\Delta \rho _{k} = \frac{b_k}{V_k} - \rho _{solvent}$, *b* and $\rho$ denoting scattering length and scattering length density for either radiation (X-rays or neutrons). A graphical representation of all distances between moieties and thicknesses is given in Fig. 4.

The full model includes the vesicle form factor $F_{sphere}$, the weighted average of bilayer form factors $F_{bil}$, which we split into real and imaginary part, and the incoherent background $I_{inc}$:

5 $$I(q)\, \propto \,F_{{{\text{sphere}}}} (R_{m} ,\sigma _{R} )\sum\limits_{k} {\mathcal{N}} \left( {D_{{C,k}} |\overline{{D_{C} }} ,\sigma _{{{\text{poly}}}} } \right)\,\left\{ {\left. {\left| {F_{{bil,k}}^{{real}} } \right|^{2} \, + \,\left| {F_{{bil,k}}^{{imag}} } \right|^{2} } \right|} \right\}\, + \,I_{{inc}}$$

with

6 $$\begin{aligned} \begin{aligned} F_{bil,k}^{real} =&4\Delta \rho _\text {bw}\frac{1}{q} \mathrm {e}^{-\frac{q^2\sigma _\text {CH2}^\text {in~2}}{2}} \sin \biggl (q\frac{d^\text {in}_\text {BB}+d^\text {in}_{PC}+d^\text {in}_{shell}}{2}\biggr ) \\&\cos \left( {q\left( { - D_{{C,k}}^{{{\text{in}}}} - \frac{{d_{{{\text{BB}}}}^{{{\text{in}}}} + d_{{PC}}^{{{\text{in}}}} d_{{shell}}^{{{\text{in}}}} }}{2}} \right)} \right) \\&+2(\Delta \rho ^\text {in}_{PC}-\Delta \rho _\text {bw}) \frac{V_{PC}}{A} \mathrm {e}^{-\frac{q^{2}{\sigma ^{\text {in}}_{PC}}^2}{2}}\\&\quad \cos \left( {q\left( {\frac{{ - D_{{C,k}}^{{{\text{in}}}} - d_{{{\text{BB}}}}^{{{\text{in}}}} - d_{{PC}}^{{{\text{in}}}} }}{2}} \right)} \right) \\&+ 2(\Delta \rho ^\text {in}_\text {BB}-\Delta \rho _\text {bw}) \frac{V^\text {in}_\text {BB}}{A} \mathrm {e}^{-\frac{q^{2}{\sigma ^{\text {in}}_{BB}}^2}{2}}\\&\quad \cos \left( {q\left( {\frac{{ - D_{{C,k}}^{{{\text{in}}}} - d_{{{\text{BB}}}}^{{{\text{in}}}} }}{2}} \right)} \right) \\&+ 2 \Delta \rho ^\text {in}_\text {CH2} \frac{1}{q}\mathrm {e}^{-\frac{q^{2}{\sigma ^\text {in}_\text {CH2}}^2}{2}}\sin \left( {\frac{{qD_{{C,k}}^{{{\text{in}}}} }}{2}} \right) \\&\quad \cos \left( {q\left( {\frac{{ - D_{{C,k}}^{{{\text{in}}}} }}{2}} \right)} \right) \\&+ f_\text {CH3,k} \mathrm {e}^{-\frac{q^{2}{\sigma _\text {CH3}}^2}{2}} \sin \left( {\frac{{qz_{{{\text{CH3}}}} }}{2}} \right) \\&\quad \cos \left( {\frac{{qz_{{{\text{CH3}}}} }}{2}} \right) \\&+ 2 \Delta \rho _\text {CH2} \frac{1}{q}\mathrm {e}^{-\frac{q^{2}{\sigma _\text {CH2}}^2}{2}}\sin \left( {\frac{{qD_{{C,k}}^{{{\text{out}}}} }}{2}} \right) \\&\quad \cos \left( {\frac{{qD_{{C,k}}^{{{\text{out}}}} }}{2}} \right) \\&+ 2(\Delta \rho ^\text {out}_\text {BB}-\Delta \rho _\text {bw}) \frac{V^\text {out}_\text {BB}}{A} \mathrm {e}^{-\frac{q^{2}{\sigma ^\text {out}_\text {BB}}^2}{2}}\\&\quad \cos \left( {q\left( {\frac{{D_{{C,k}}^{{{\text{out}}}} + d_{{{\text{BB}}}}^{{{\text{out}}}} }}{2}} \right)} \right) \\&+ 2(\Delta \rho ^\text {out}_{PC}-\Delta \rho _\text {bw}) \frac{V^\text {out}_{PC}}{A} \mathrm {e}^{-\frac{q^{2}{\sigma ^\text {out}_{PC}}^2}{2}}\\&\quad \cos \left( { q\left( {\frac{{D_{{C,k}}^{{{\text{out}}}} + d_{{{\text{BB}}}}^{{{\text{out}}}} + d_{{PC}}^{{{\text{out}}}} }}{2}} \right) } \right) \\&+ 4\Delta \rho _\text {bw}\frac{1}{q} \mathrm {e}^{-\frac{q^{2}{\sigma ^\text {out}_\text {CH2}}^2}{2}}\sin \left( {q\frac{{d_{{{\text{BB}}}}^{{{\text{out}}}} + d_{{PC}}^{{{\text{out}}}} + d_{{shell}}^{{{\text{out}}}} }}{2}} \right) \\&\cos \left( {q\left( {D_{{C,k}}^{{{\text{out}}}} + \frac{{d_{{{\text{BB}}}}^{{{\text{out}}}} + d_{{PC}}^{{{\text{out}}}} + d_{{shell}}^{{{\text{out}}}} }}{2}} \right)} \right) \end{aligned} \end{aligned}$$

and

7 $$\begin{aligned} \begin{aligned} F_{bil,k}^{imag} =&4\Delta \rho _\text {bw}\frac{1}{q} \mathrm {e}^{-\frac{q^{2}\sigma _\text {CH2}^\text {in~2}}{2}} \sin \biggl (q\frac{d^\text {in}_\text {BB}+d^\text {in}_{PC}+d^\text {in}_{shell}}{2}\biggr )\\&\sin \left( {q\left( { - D_{{C,k}}^{{{\text{in}}}} - \frac{{d_{{{\text{BB}}}}^{{{\text{in}}}} + d_{{PC}}^{{{\text{in}}}} d_{{shell}}^{{{\text{in}}}} }}{2}} \right)} \right) \\&\quad +2(\Delta \rho ^\text {in}_{PC}-\Delta \rho _\text {bw}) \frac{V_{PC}}{A} \mathrm {e}^{-\frac{q^{2}{\sigma ^\text {in}_{PC}}^2}{2}}\\&\quad \sin \left( {q\left( {\frac{{ - D_{{C,k}}^{{{\text{in}}}} - d_{{{\text{BB}}}}^{{{\text{in}}}} - d_{{PC}}^{{{\text{in}}}} }}{2}} \right)} \right) \\&\quad + 2(\Delta \rho ^\text {in}_\text {BB}-\Delta \rho _\text {bw}) \frac{V^\text {in}_\text {BB}}{A} \mathrm {e}^{-\frac{q^{2}{\sigma ^\text {in}_\text {BB}}^2}{2}}\\&\quad \sin \left( {q \left( {\frac{{ - D_{{C,k}}^{{{\text{in}}}} - d_{{{\text{BB}}}}^{{{\text{in}}}} }}{2}} \right) } \right) \\&\quad + 2 \Delta \rho ^\text {in}_\text {CH2} \frac{1}{q}\mathrm {e}^{-\frac{q^{2}{\sigma ^\text {in}_\text {CH2}}^2}{2}}\sin \left( {\frac{{qD_{{C,k}}^{{{\text{in}}}} }}{2}} \right) \\&\quad \sin \left( {\frac{{q( - D_{{C,k}}^{{{\text{in}}}} }}{2}} \right) \\&\quad + f_\text {CH3,k} \mathrm {e}^{-\frac{q^{2}{\sigma _\text {CH3}}^2}{2}} \sin \left( {\frac{{qz_{{{\text{CH3}}}} }}{2}} \right) \\&\quad \sin \left( {\frac{{qz_{{{\text{CH3}}}} }}{2}} \right) \\&\quad + 2 \Delta \rho _\text {CH2} \frac{1}{q}\mathrm {e}^{-\frac{q^{2}{\sigma _\text {CH2}}^2}{2}}\sin \left( {\frac{{qD_{{C,k}}^{{{\text{out}}}} }}{2}} \right) \\&\quad \sin \left( {\frac{{qD_{{C,k}}^{{{\text{out}}}} }}{2}} \right) \\&\quad + 2(\Delta \rho ^\text {out}_\text {BB}-\Delta \rho _\text {bw}) \frac{V^\text {out}_\text {BB}}{A} \mathrm {e}^{-\frac{q^{2}{\sigma ^\text {out}_\text {BB}}^2}{2}}\\&\quad \sin \left( { q\left( {\frac{{D_{{C,k}}^{{{\text{out}}}} + d_{{{\text{BB}}}}^{{{\text{out}}}} }}{2}} \right) } \right) \\&\quad + 2(\Delta \rho ^\text {out}_{PC}-\Delta \rho _\text {bw}) \frac{V^\text {out}_{PC}}{A} \mathrm {e}^{-\frac{q^{2}{\sigma ^\text {out}_{PC}}^2}{2}}\\&\quad \sin \left( { q\left( {\frac{{D_{{C,k}}^{{{\text{out}}}} + d_{{{\text{BB}}}}^{{{\text{out}}}} + d_{{PC}}^{{{\text{out}}}} }}{2}} \right) } \right) \\&\quad + 4\Delta \rho _\text {bw}\frac{1}{q} \mathrm {e}^{-\frac{q^{2}{\sigma ^\text {out}_\text {CH2}}^2}{2}} \sin \left( {q\frac{{d_{{{\text{BB}}}}^{{{\text{out}}}} + d_{{PC}}^{{{\text{out}}}} + d_{{shell}}^{{{\text{out}}}} }}{2}} \right) \\&\sin \left( {q\left( {D_{{C,k}}^{{{\text{out}}}} + \frac{{d_{{{\text{BB}}}}^{{{\text{out}}}} + d_{{PC}}^{{{\text{out}}}} + d_{{shell}}^{{{\text{out}}}} }}{2}} \right)} \right) \end{aligned} \end{aligned}$$

The prefactor for the meythyl group error functions $f_\text {CH3,k}$ is given by:

8 $$f_{{{\text{CH3,k}}}} \, = \,\left[ {\frac{{\Delta \rho _{{{\text{CH3}}}}^{{{\text{in}}}} + \Delta \rho _{{{\text{CH3}}}}^{{{\text{out}}}} }}{2} - \frac{{\left( {1 + \frac{{V_{s} }}{{V_{{{\text{CH3}}}}^{{{\text{in}}}} }}} \right)}}{{2\,\Delta \rho _{{{\text{CH2}}}}^{{{\text{in}}}} }}{\mkern 1mu} - \frac{{\left( {1 - \frac{{V_{s} }}{{V_{{{\text{CH3}}}}^{{{\text{out}}}} }}} \right)}}{{2\,\Delta \rho _{{{\text{CH2}}}}^{{{\text{out}}}} }}} \right]{\mkern 1mu} \frac{{2(V_{{{\text{CH3}}}}^{{{\text{in}}}} + V_{{{\text{CH3}}}}^{{{\text{out}}}} )}}{{\left( {1 + \frac{{V_{s} }}{{V_{{{\text{CH3}}}}^{{{\text{out}}}} }}} \right)\frac{{V_{{{\text{CH3}}}}^{{{\text{in}}}} + V_{{{\text{CH2}}}}^{{{\text{in}}}} + V_{s} }}{{D_{{C,k}}^{{{\text{in}}}} }} + \left( {1 - \frac{{V_{s} }}{{V_{{{\text{CH3}}}}^{{{\text{in}}}} }}} \right)\frac{{V_{{{\text{CH3}}}}^{{{\text{out}}}} + V_{{{\text{CH2}}}}^{{{\text{out}}}} - V_{s} }}{{D_{{C,k}}^{{{\text{out}}}} }}}}$$

To describe the contribution from the overall vesicle shape we use the Schultz-distributed form factor of a sphere, as described in Kučerka et al. (2007):

9 $$F_{{{\text{sphere}}}} \, = \,\frac{{8\pi ^{2} (z + 1)(z + 2)}}{{s^{2} q^{2} }}\left\{ {1 - \left( {1 + \frac{{4q^{2} }}{{s^{2} }}} \right)^{{ - (z + 3)/2}} \,\cos \left[ {(z + 3)\arctan \left( {\frac{{2q}}{s}} \right)} \right]} \right\}$$

Mean vesicle radius $R_m$ and polydispersity $\sigma _R$ enter via the auxiliary quantities $s= \frac{R_m}{\sigma _R^2}$ and $z= \frac{R_m^2}{\sigma _R^2}-1$.

## Stability of Membrane Asymmetry

To check the stability of our asymmetric vesicles, we monitored their SANS signal over one night while keeping them at 50 °C. The systems DPPC$^\text {in}$/POPC$^\text {out}$, DPPC$^\text {in}$/SOPC$^\text {out}$, DPPC$^\text {in}$/MSPC$^\text {out}$ and DPPC$^\text {in}$/SMPC$^\text {out}$ were completely stable both in their overall vesicle structure (low-*q*) and in their transmembrane structure (high-*q*). For DPPC$^\text {in}$/PMPC$^\text {out}$ the low-*q* region also remained unchanged. However, in the high-*q*, the sample slowly equilibrated towards a less asymmetric state (Fig. 6). We hypothesize that the reason we could only observe this flip–flop for the PMPC-donor is due to its lower hydrophobic volume and thus a lower energy barrier for the head-groups when traversing through the membrane. Compared to the timescale of our other SAS-experiments, the flip–flop was slow and is therefore assumed not to interfere with the results.

## Code Availability

Upon request (M.P.K.F. and G.P.)
