# Supplementary material for: Interdigitation-Induced Order and Disorder in Asymmetric Membranes
Source: J Membr Biol. 2022 Apr 26;255(4-5):407–21. doi: 10.1007/s00232-022-00234-0 (PMC9581838; doi:10.1007/s00232-022-00234-0)
Supplement: Supplementary file 1 — Supplementary file1 (PDF 1286 kb) [file 232_2022_234_MOESM1_ESM.pdf]

Interdigitation-induced Order and Disorder in Asymmetric Membranes - Supplementary Information

Moritz PK Frewein, Paulina Piller, Enrico F Semeraro, Krishna C Batchu, Frederick A Heberle, Haden L Scott, Yuri Gerelli, Lionel Porcar and Georg Pabst

1 SAS-model parameters of pure lipids

Table S1: Properties of pure lipid bilayers as published in [1]. Bilayer thicknesses have been updated. The lower part of the table contains parameters used in fitting aLUV SAS data. These were taken from [2] and fixed during the analysis.

	DPPC	MSPC	SMPC	PMPC	MSM	POPC	SOPC
D_B [Å]	39.0	39.6	39.7	37.4	41.3	37.83	38.73
$D_B/2$ [Å]	19.5	19.8	19.9	18.7	20.6	18.9	19.4
D_C [Å]	14.3	14.5	14.6	13.5	16.4	14.18	15.9
A [Å^2]	63.1	62.2	62.0	62.9	64.8	67.5	68.8
σ_{poly} %	3.6	2.9	5.3	3.5	3.5	7.9	3.6
V_W [Å^3]	29.3	29.3	29.3	29.3	29.8	29.9	29.7
n_W	9.7	11.3	12.8	12.1	9.6	16.64	15.1
V_L [Å^3]	1232	1232	1232	1175.8	1336.3	1276.9	1333.1
V_H [Å^3]	328	328	328	328	274	320	328
r_{BB}	0.44	0.44	0.44	0.44	0.32	0.45	0.44
r_{PCN}	0.3	0.3	0.3	0.3	0.32	0.29	0.3
r	2.09	2.09	2.09	2.09	2.09	2.09	2.09
r_{12}					0.8	0.8	0.8

V_L... Total lipid volume
V_H... Headgroup volume
r_{BB}... Backbone volume fraction relative to the headgroup (V_{BB}/V_H)
r_{PCN}... Phosphate group volume fraction relative to the headgroup (V_{PCN}/V_H)
r... Volume of a CH_3-segment relative to CH_2 (V_{CH3}/V_{CH2})
r_{12}... Volume of a CH-segment relative to CH_2 (V_{CH}/V_{CH2})

2 SAS-model parameters of asymmetric vesicles

Table S2: SAS-fitting parameters of aLUVs containing DPPCd62 as acceptor lipid and MSPC, SMPC, MSM, POPC and SOPC donor lipids.

	ϵ [%]	MSPC	SMPC	PMPC	MSM	POPC	SOPC
Total acc/don %	5	69:31	59:41	62:38	54:46	64:36	61:39
In acc/don %	5	96:4	92:8	95:5	83:17	84:16	93:7
Out acc/don %	5	45:55	30:70	33:67	28:72	46:54	33:67
D_B [Å]	3	37.0	38.1	36.1	40.9	36.7	37.3
D_{HH} [Å]	3	37.9	39.1	36.9	35.8	37.8	37.9
D_M^{in} [Å]	6	18.0	18.5	18.0	20.0	18.1	18.2
D_M^{out} [Å]	6	18.9	19.6	18.1	21.0	18.6	19.1
D_C^{in} [Å]	5	13.2	13.5	13.2	14.8	13.3	13.4
D_C^{out} [Å]	5	14.0	14.5	13.2	16.4	13.8	14.3
D_{H1}^{in} [Å]	20	2.6	2.9	2.5	2.7	8.3	2.5
D_{H1}^{out} [Å]	20	8.1	8.2	8.0	1.9	2.5	7.8
A^{in} [Å^2]	5	67.4	65.8	67.5	60.9	67.6	67.7
A^{out} [Å^2]	5	65.8	63.5	66.5	63.9	68.2	68.5
z_{BB}^{in} [Å]	6	-14.1	-14.4	-14.1	-15.7	-14.2	-14.3
z_{BB}^{out} [Å]	6	14.9	15.4	14.1	17.3	14.7	15.2
$\sigma_{BB}^{in/out}$ [Å]		2.1	2.1	2.1	2.1	2.1	2.1
z_{PCN}^{in} [Å]	10	-16.3	-16.8	-16.1	-18.0	-21.6	-16.2
z_{PCN}^{out} [Å]	10	22.2	22.9	21.3	20.2	16.6	22.1
σ_{PCN}^{in} [Å]	10	2.2	2.0	2.0	2.0	2.5	2.0
σ_{PCN}^{out} [Å]	10	3.1	3.3	2.8	3.6	2.0	2.0
$z_{CholCH3}^{in}$ [Å]	10	-19.3	-18.5	-19.1	-21.0	-22.6	-19.2
$z_{CholCH3}^{out}$ [Å]	10	23.2	23.9	22.3	23.2	19.6	25.1
$\sigma_{CholCH3}^{in/out \dagger}$ [Å]		3.0	3.0	3.0	3.0	3.0	3.0
$\sigma_{HC}^{in/out}$ [Å]		2.5	2.5	2.5	2.5	2.5	2.5
z_{CH3} [Å]	10	-1.0	-1.0	-0.7	-2.6	1.0	-0.4
σ_{CH3} [Å]	20	3.0	3.0	3.0	3.0	3.3	2.7
σ_{poly} [%]	6	4.1	2.8	3.3	7.3	4.4	6.1
$V_{W,bound}$ [Å^3]	6	29.4	29.3	29.6	29.5	29.6	29.6
n_W^{in}	6	9.5	6.5	9.0	8.0	16.8	9.0
n_W^{out}	6	15.7	15.2	15.8	11.1	9.0	20.4
R_m [Å]	10	400	340	360	360	390	390
σ_R [Å]	10	110	90	90	120	100	100

D_{H1}... distance between head group (phosphate) and hydrophobic/hydrophilic interface
$z_x^{in/out}$... position of moiety x relative to the membrane center
$\sigma_x^{in/out}$... Gaussian standard deviation of moiety x
R_m... mean vesicle radius
σ_R... vesicle radius polydispersity

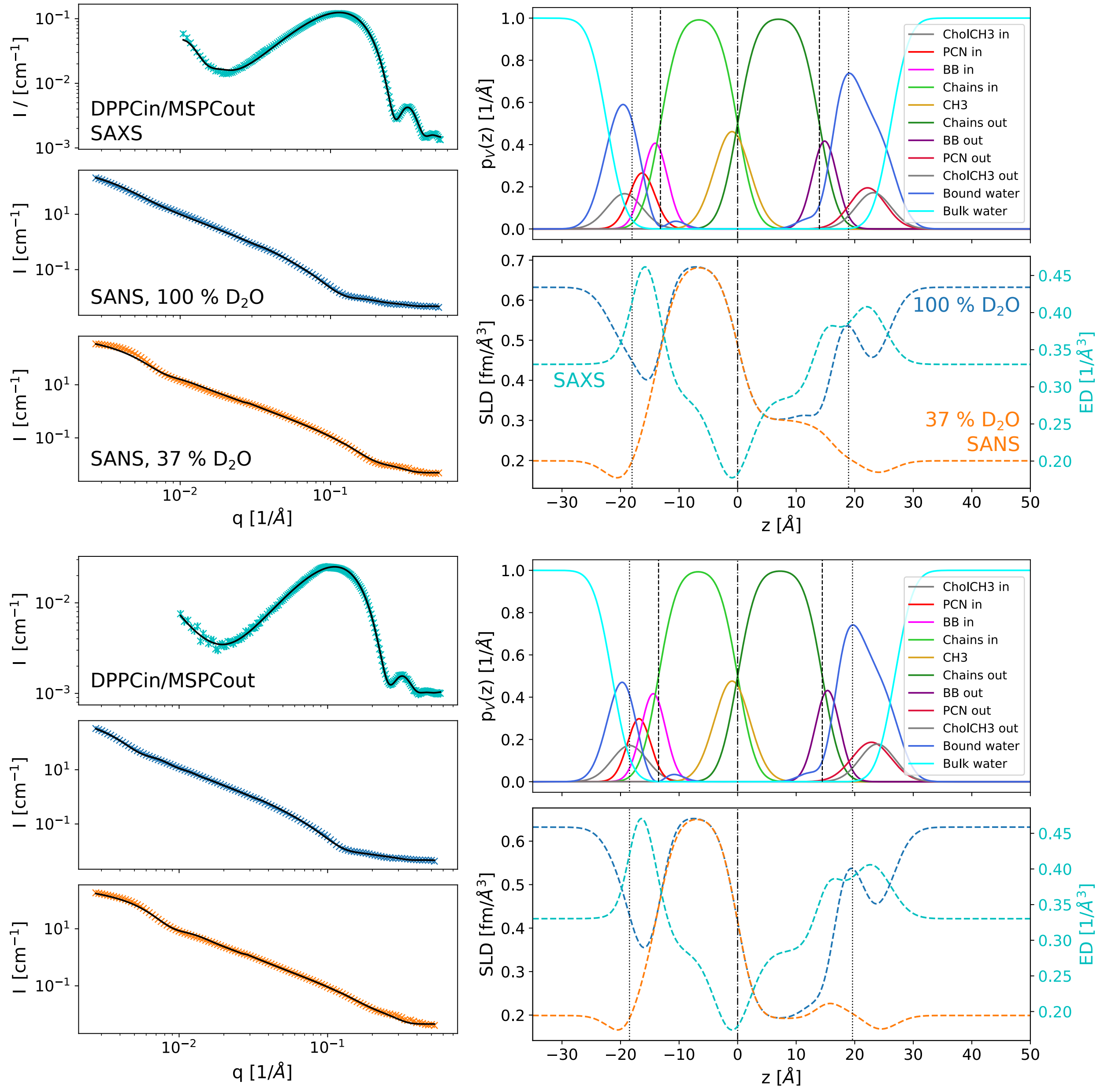

Figure S1: SAXS and SANS data with fits (black lines); SDP volume probability, electron density and neutron scattering length density profiles for the systems DPPC^{in}/MSPC^{out} and DPPC^{in}/SMPC^{out}.

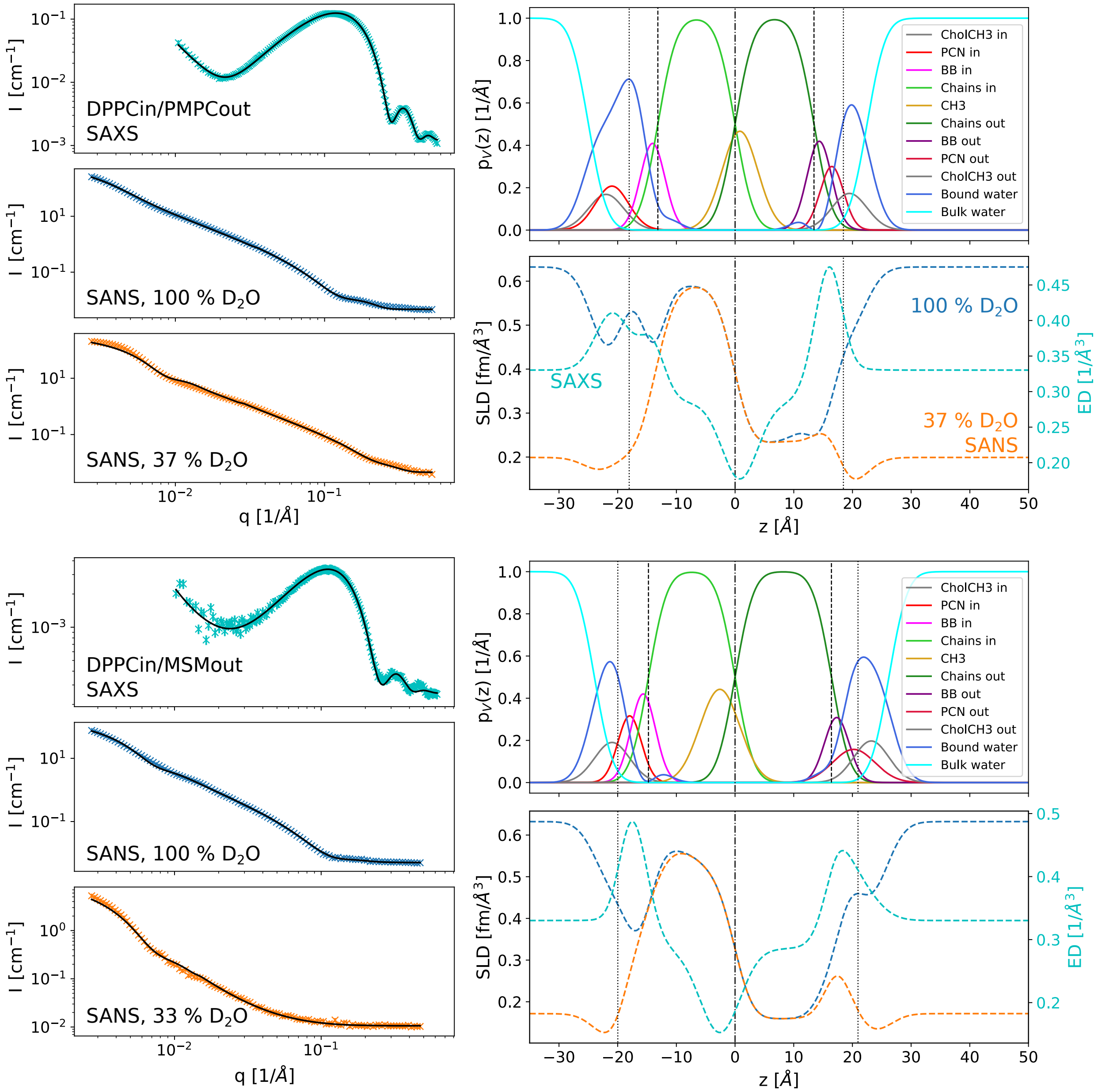

Figure S2: SAXS and SANS data with fits (black lines); SDP volume probability, electron density and neutron scattering length density profiles for the systems DPPC^{in}/PMPC^{out} and DPPC^{in}/MSMout.

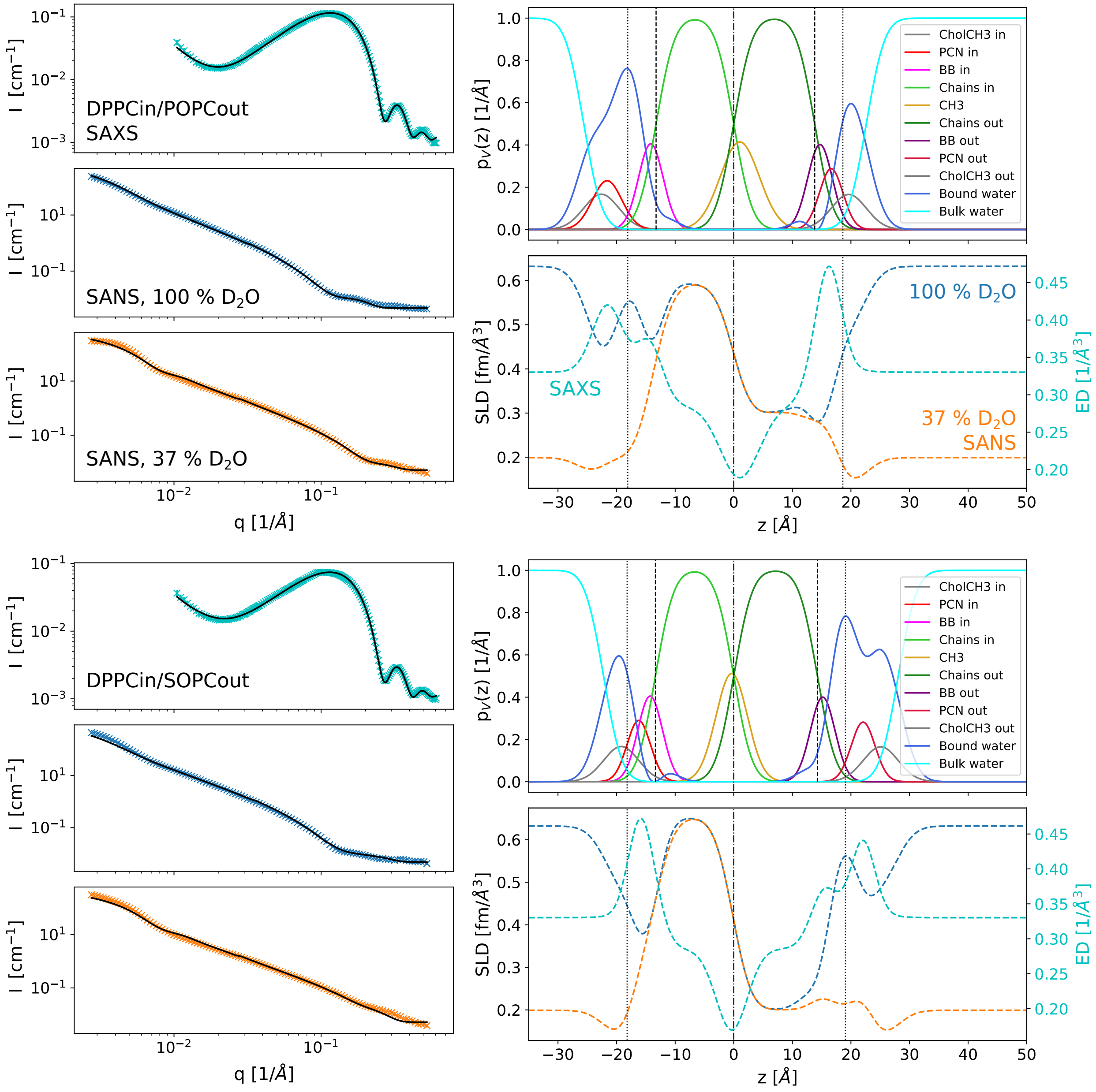

Figure S3: SAXS and SANS data with fits (black lines); SDP volume probability, electron density and neutron scattering length density profiles for the systems $DPPC^{in}/POPC^{out}$ and $DPPC^{in}/SOPC^{out}$.

3 SAS-model parameters of reference LUVs

Table S3: SAS-fitting parameters of symmetric reference LUVs containing 90% (in) or 30% (out) DPPC, complemented by MSPC, SMPC, or PMPC.

		MSPC		SMPC		PMPC	
	ϵ [%]	in	out	in	out	in*	out
D_B [Å]	3	39.9	39.6	39.7	39.30	38.53	38.66
D_{HH} [Å]	3	35.3	35.1	35.1	34.78	34.77	33.92
$2D_C$ [Å]	3	29.3	29.1	29.1	28.84	28.22	28.03
D_{H1} [Å]	20	3.0	3.0	3.0	3.0	3.3	2.9
A [Å^2]	2	61.7	62.2	62.1	62.7	63.7	61.7
z_{BB} [Å]	8	16.3	15.9	16.0	15.8	17.1	15.7
σ_{BB} [Å]	20	2.5	2.5	2.5	2.5	2.5	2.5
z_{PCN} [Å]	8	18.3	18.3	18.2	18.2	17.2	17.5
σ_{PCN} [Å]	20	3.5	3.3	3.5	3.5	2.2	3.5
$z_{CholCH3}$ [Å]	3	18.8	19.0	20.1	19.3	22.2	18.6
$\sigma^{\dagger}_{CholCH3}$ [Å]		3.0	3.0	3.0	3.0	3.0	3.0
σ_{HC} [Å]		2.5	2.5	2.5	2.5	2.5	2.5
σ_{CH3} [Å]	5	2.7	3.2	3.6	3.5	2.4	3.0
σ_{poly} [%]	6	4.0	4.0	8.4	4.7	0.0	3.7
$V_{W,bound}$ [Å^3]	6	27.3	27.4	27.3	27.5	27.3	27.5
n_W	6	3.7	4.6	6.8	5.5	12.5	4.5
R_m [Å]	10	480	340	350	440	420	470
σ_R [Å]	10	130	200	130	140	170	160

Table S4: SAS-fitting parameters of symmetric reference LUVs containing 90% (in) or 30% (out) DPPC, complemented by MSM, POPC, or SOPC.

		MSM		POPC		SOPC	
	ϵ [%]	in	out	in	out	in	out
D_B [Å]	3	38.7	41.29	39.4	38.4	39.9	38.7
D_{HH} [Å]	3	38.4	40.79	34.8	34.6	35.1	35.5
$2D_C$ [Å]	3	28.7	32.1	29.0	28.4	29.4	29.0
D_{H1} [Å]	20	4.87	4.35	2.9	3.1	2.87	3.26
A [Å^2]	2	64.19	63.22	62.8	65.9	62.25	67.32
z_{BB} [Å]	8	15.21	17.28	15.7	16.1	16.27	16.68
σ_{BB} [Å]	20	2.5	2.5	2.5	2.5	2.5	2.5
z_{PCN} [Å]	8	20.04	20.9	18.3	17.7	18.2	18.27
σ_{PCN} [Å]	20	2.97	2.72	3.4	3.4	3.5	3.25
$z_{CholCH3}$ [Å]	3	20.04	20.9	22.0	18.4	20.46	22.9
$\sigma^{\dagger}_{CholCH3}$ [Å]		3	3	3.0	3.0	3	3
σ_{HC} [Å]		2.5	2.5	2.5	2.5	2.5	2.5
σ_{CH3} [Å]	5	4	4	3.1	3.5	3.09	3.14
σ_{poly} [%]	6	3.91	6.19	3.6	6.9	4.75	4.21
$V_{W,bound}$ [Å^3]	6	28.28	28.68	29.0	27.3	28.21	29.28
n_W	6	7.83	6.81	11.0	4.9	7.22	14.58
R_m [Å]	10	430	500	470	480	520	540
σ_R [Å]	10	120	150	190	170	190	170

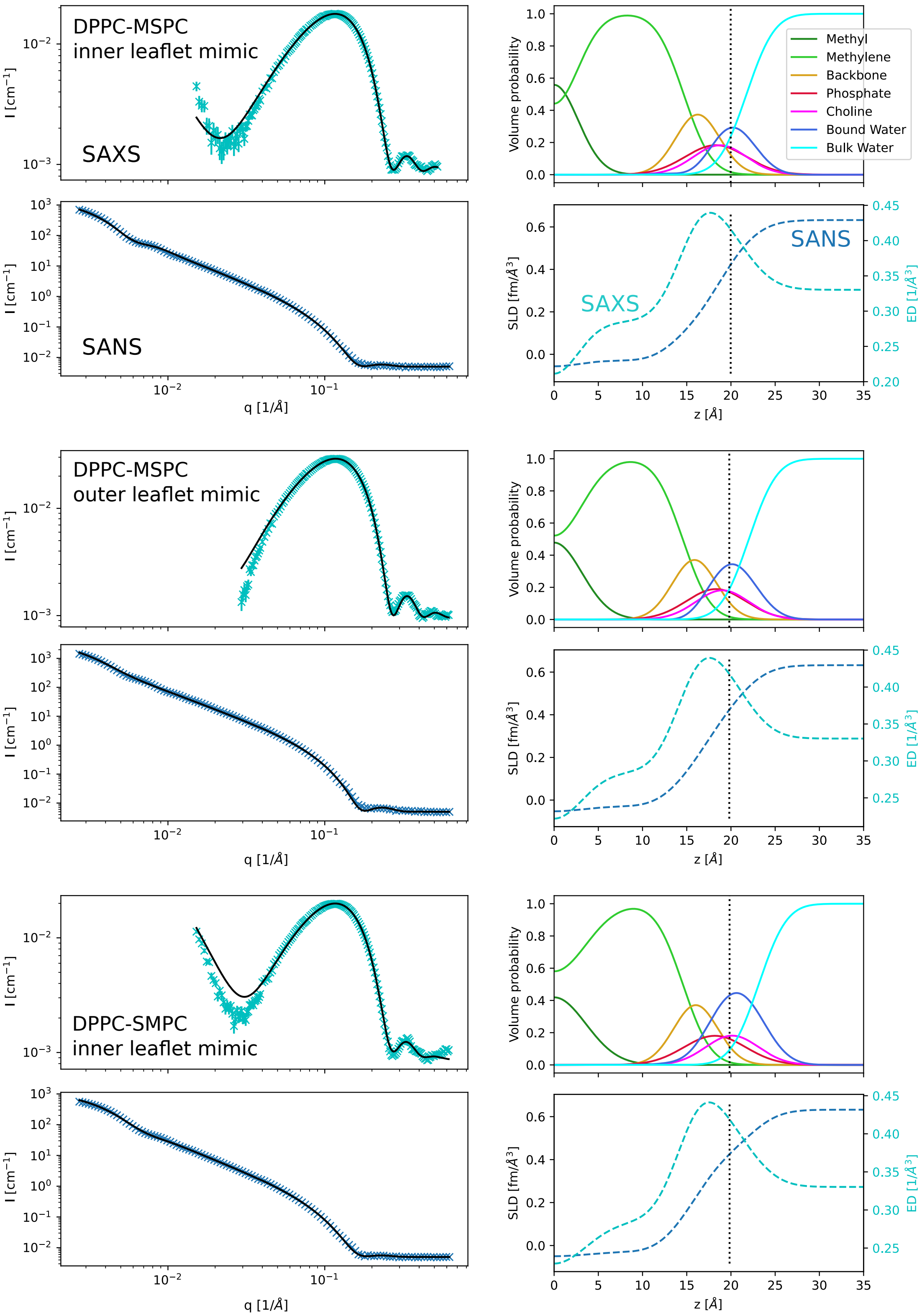

Figure S4: SAXS and SANS data with fits (black lines); SDP volume probability, electron density and neutron scattering length density profiles for DPPC-MSPC inner/outer leaflet symmetric mimics, as well as DPPC-SMPC inner leaflet mimics.

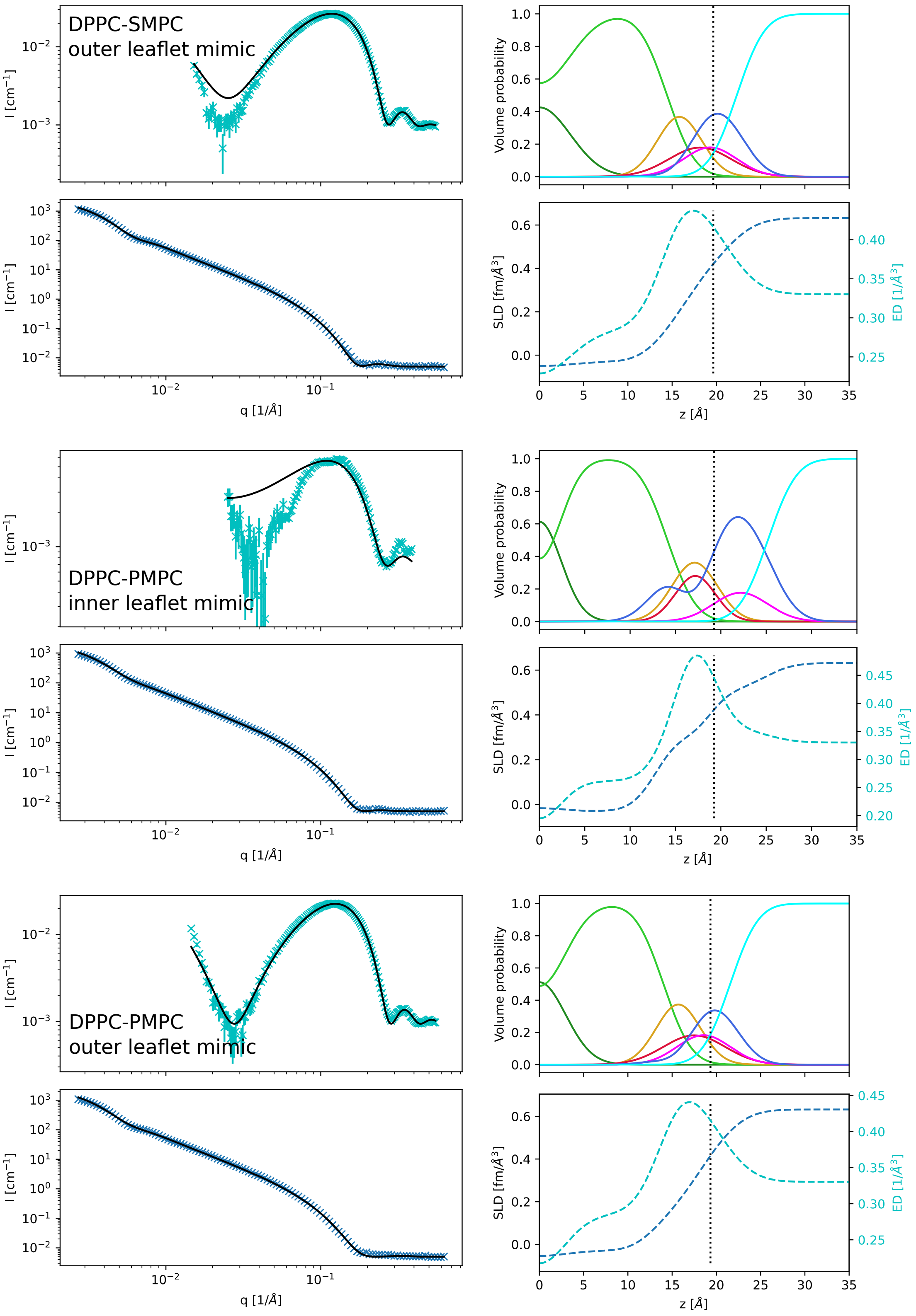

Figure S5: SAXS and SANS data with fits (black lines); SDP volume probability, electron density and neutron scattering length density profiles for DPPC-SMPC outer leaflet mimics and DPPC-PMPC inner/outer leaflet symmetric mimics.

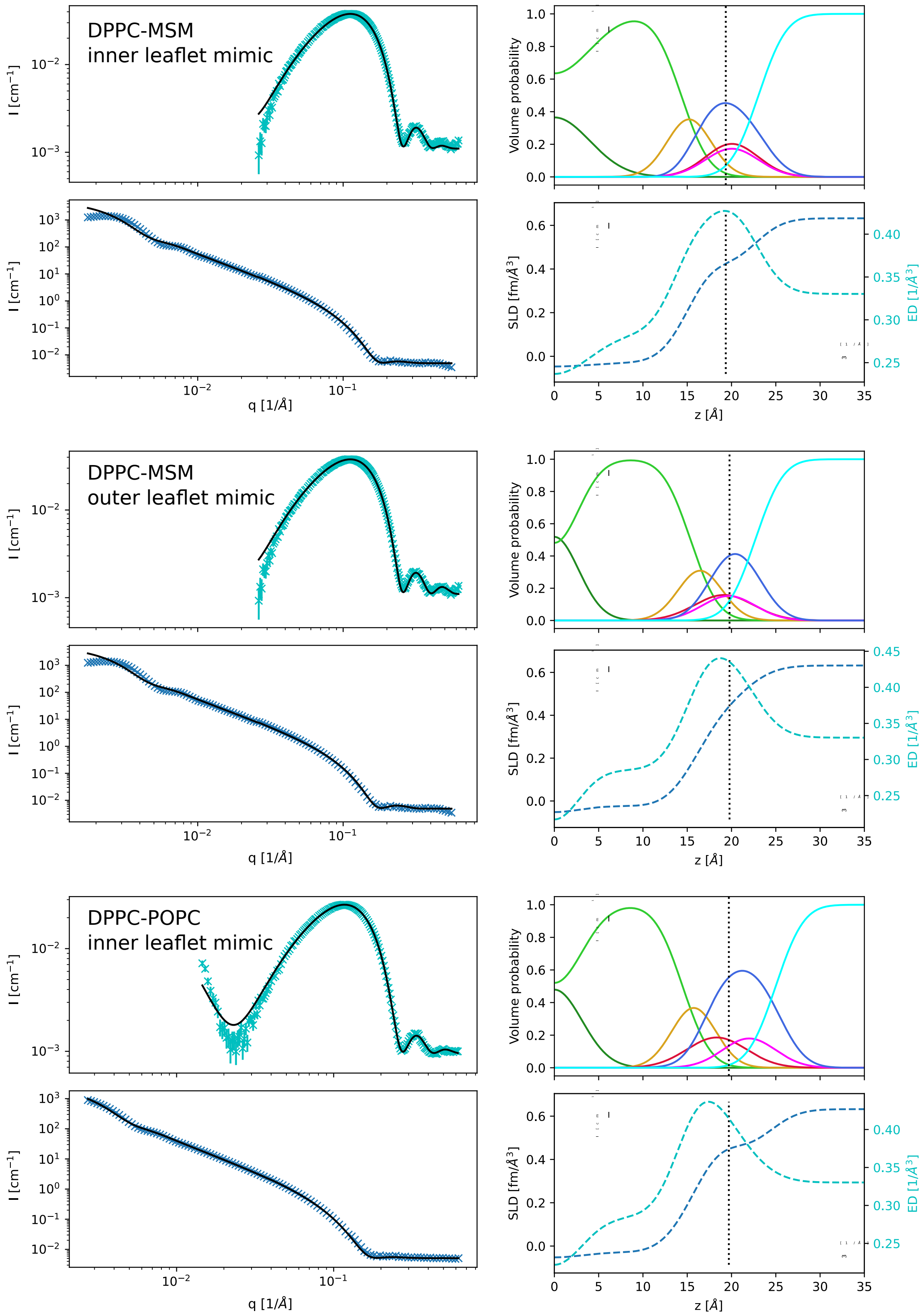

Figure S6: SAXS and SANS data with fits (black lines); SDP volume probability, electron density and neutron scattering length density profiles for DPPC-MSM inner/outer leaflet symmetric mimics, as well as DPPC-POPC inner leaflet mimics.

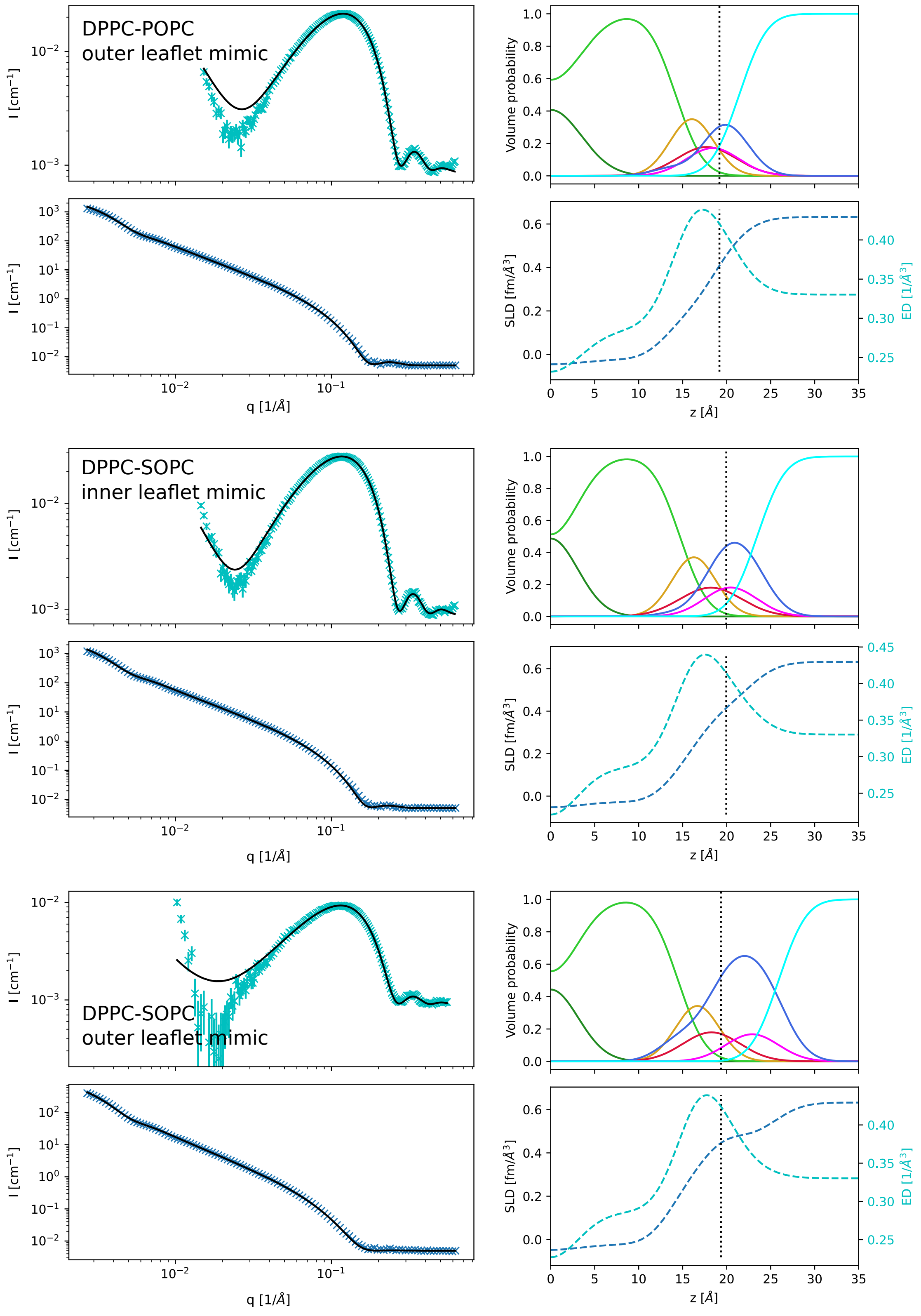

Figure S7: SAXS and SANS data with fits (black lines); SDP volume probability, electron density and neutron scattering length density profiles for DPPC-POPC outer leaflet mimics and DPPC-SOPC inner/outer leaflet symmetric mimics.

4 Gas Chromatography

Table S5: Calibration curves of all hydrocarbon chains occurring in the used lipids and the 3 most abundant chains in MSM. In the last line the slope k of the linear fit is given.

μmol	C14:0	μmol	C16:0d	μmol	C16:0	μmol	C18:0
0.002	19747	0.002	16043	0.002	17697	0.002	15594
0.004	36066	0.004	36370	0.004	31270	0.004	33143
0.015	110938	0.013	87194	0.014	114122	0.013	106044
0.030	276664	0.025	238040	0.027	266841	0.025	241047
0.059	596070	0.050	528481	0.055	558479	0.051	567215
0.118	994664	0.101	1064457	0.109	1064457	0.101	1079925
0.236	2046908	0.201	2768564	0.218	2423189	0.203	1877413
0.472	3768187	0.402	3779355	0.436	3600379	0.405	4021069
k [mol/count]	1.14E-07		9.31E-08		1.03E-07		9.99E-08

μmol	C18:1	μmol	MSM C22:0	C23:0	C24:0
0.002	20803				
0.004	42162				
0.012	97706	0.013	5917	7228	6132
0.025	257572	0.025	13873	19961	13672
0.049	557266	0.051	30440	45868	30504
0.099	1064919	0.102	45564	69095	46701
0.198	2824137	0.204	115858	176708	116561
0.395	3773470	0.408	126005	181364	123150
k [mol/count]	8.94E-08		2.28E-06	1.49E-06	2.28E-06

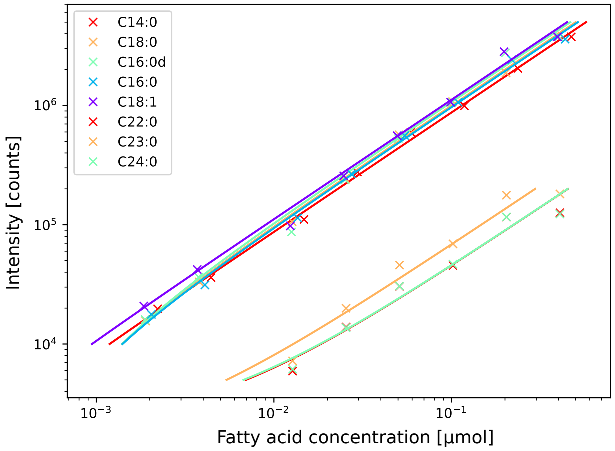

Figure S8: Calibration curves as given in tab. S5 and linear fits.

Table S6: Results from GC-analysis of asymmetric vesicles.

DPPC^{in}/MSPC^{out}						
	C16:0d	C14:0	C18:0	χ_{acc}	χ_{don}	
Sample 1	205104	58523	41678			
	0.020	0.007	0.005	0.63	0.38	
Sample 2	215107	61004	43908			
	0.021	0.007	0.005	0.64	0.36	
DPPC^{in}/SMPC^{out}						
	C16:0d	C14:0	C18:0	χ_{acc}	χ_{don}	
Sample 1	409420	158486	125415			
	0.039	0.016	0.014	0.57	0.43	
Sample 2	457413	177140	139695			
	0.043	0.018	0.016	0.56	0.44	
DPPC^{in}/PMPC^{out}						
	C16:0d	C16:0	C14:0	χ_{acc}	χ_{don}	
Sample 1	1590184	768008	643108			
	0.149	0.079	0.074	0.49	0.51	
Sample 2	1616790	780218	654394			
	0.151	0.081	0.075	0.49	0.51	
DPPC^{in}/POPC^{out}						
	C16:0d	C16:0	C18:1	χ_{acc}	χ_{don}	
Sample 1	409420	158486	125415			
	0.029	0.011	0.009	0.59	0.41	
Sample 2	457413	177140	139695			
	0.032	0.013	0.010	0.58	0.42	
DPPC^{in}/SOPC^{out}						
	C16:0d	C18:0	C18:1	χ_{acc}	χ_{don}	
Sample 1	213421	46984	67409			
	0.020	0.005	0.006	0.65	0.35	
Sample 2	234851	50921	72924			
	0.022	0.005	0.007	0.65	0.35	
DPPC^{in}/MSMout						
	C16:0d	C22:0	C23:0	C24:0	χ_{acc}	χ_{don}
Sample 1	213421	17259	26170	18423		
	0.049	0.035	0.037	0.037	0.57	0.43

References

[1] Frewein, M.P.K., Doktorova, M., Heberle, F.A., Scott, H.L., Semeraro, E.F., Porcar, L., Pabst, G.: Structure and Interdigitation of Chain-Asymmetric Phosphatidylcholines and Milk Sphingomyelin in the Fluid Phase. Symmetry **13**(8), 1441 (2021). https://doi.org/10.3390/sym13081441

[2] Nagle, J.F., Venable, R.M., Maroclo-Kemmerling, E., Tristram-Nagle, S., Harper, P.E., Pastor, R.W.: Revisiting Volumes of Lipid Components in Bilayers. Journal of Physical Chemistry B **123**(12), 2697–2709 (2019). https://doi.org/10.1021/acs.jpcb.8b12010
